# Synthesis and photoluminescence of iridium(iii) arylacetylide complexes with acetylide-localized emissive excited states

**DOI:** 10.1039/d5dt02734a

**Published:** 2026-01-27

**Authors:** Son N. T. Phan, João V. Schober, Judy I. Wu, Thomas S. Teets

**Affiliations:** a Department of Chemistry, University of Houston 3585 Cullen Blvd Room 112 Houston TX 77204-5003 USA tteets@uh.edu

## Abstract

Herein, we report a series of three bis-cyclometalated iridium(iii) complexes each bearing an arylacetylide and an N-heterocyclic carbene (NHC) ligand. The cyclometalating ligand is 2-(2,4-difluorophenyl)pyrazole (F_2_ppz), chosen to ensure the 5d(Ir) → π*(F_2_ppz) ^3^MLCT excited-state is higher in energy than the ^3^(π → π*) state of the acetylide, allowing luminescence to originate from the latter. The strong-field NHC is included to alleviate photoluminescence (PL) quenching caused by the thermal population of the triplet metal-centered (^3^MC) states, but nevertheless, the three compounds are weakly luminescent. The parent phenylacetylide complex only emits blue luminescence in dichloromethane solution at 77 K, with a peak wavelength of 424 nm. Complexes bearing substituted phenylacetylides that engender emission in the green and yellow regions are also not emissive in solution at room temperature, but phosphoresce weakly in poly(methylmethacrylate) film and also emit at 77 K in solution. Since the emissive excited states in these compounds are acetylide-localized, we tested whether cyclometalating ligands are needed at all by preparing a new 1,2,3,4,5-pentamethylcyclopentadienyl Ir(iii) acetylide complex, but this complex does not luminesce under any of the conditions tested. The four reported complexes are structurally characterized by multinuclear NMR and one of them by single-crystal X-ray diffraction. Thorough electrochemical and photophysical studies of the complexes were also carried out, complemented by time-dependent density functional theory (TD-DFT) calculations.

## Abstract 2

Two types of Ir(iii) acetylide complexes are reported. The cyclometalated complexes exhibit weak luminescence from a localized ^3^(π → π*) state on the arylacetylide, whereas the pentamethylcyclopentadienyl-supported complex is not luminescent.

## Graphical abstract

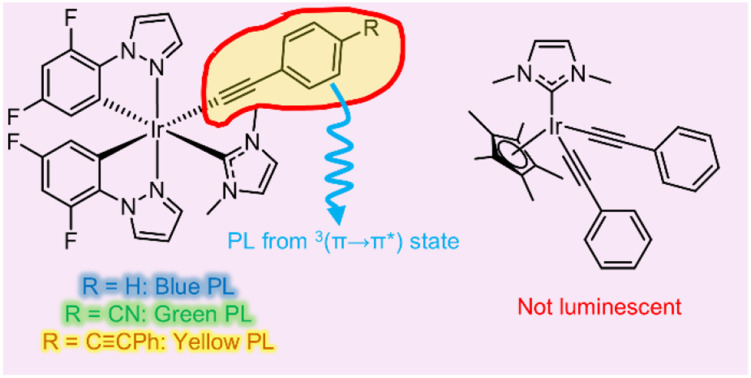

## Introduction

Cyclometalated iridium(iii) complexes are among the most extensively studied class of molecular phosphors due to their numerous applications, including photoredox catalysis,^1–3^ bioimaging,^4–6^ sensing,^7,8^ and organic light-emitting diodes (OLEDs).^9–11^ For OLED applications, the strong Ir-induced spin–orbit coupling (SOC) gives rise to fast radiative rates, high photoluminescence quantum yields, and relatively short lifetimes,^12,13^ making these complexes ideal materials for OLED dopants over the entire visible spectrum, with further improvements in blue-emitting complexes particularly sought-after.^14–19^ The emissive excited state in most cyclometalated iridium complexes can be described in simple terms as a triplet metal-to-ligand charge transfer (^3^MLCT) state involving the Ir filled 5dπ and an empty π* LUMO on a cyclometalating ligand. For blue phosphorescence, this requires cyclometalating ligands that can engender large HOMO–LUMO gaps, and there are many homoleptic cyclometalated Ir(iii) complexes with a few different types of cyclometalating ligands^20^ that have been studied extensively in the context of blue phosphorescence.^21–24^ Meanwhile, strong-field ancillary ligands, such as isocyanides, cyanide, and N-heterocyclic carbenes (NHCs), have been used in heteroleptic bis-cyclometalated Ir(iii) complexes to effectively suppress the nonemissive relaxation pathways caused by the thermal population of triplet metal-centered (^3^MC) states. As such, blue-emitting heteroleptic Ir complexes bearing chromophoric cyclometalating ligands and supported by strong-field ancillary ligands have been well investigated.^9,25–27^

Besides cyclometalating ligands, arylacetylides also represent a common class of conjugated ligands that can engender blue phosphorescence in organometallic compounds. When acetylides serve as chromophoric ligands, the emissive excited states tend to be more localized, ^3^(π → π*) in nature, with less pronounced participation of metal d orbitals. This results in sharp photoluminescence bands and engenders facile color tuning through modification of the arylacetylide, features that have been leveraged in a few different classes of blue-phosphorescent platinum(ii) acetylide complexes.^28–30^ A limitation of most Pt(ii) acetylide complexes is small radiative rate constants (*k*_r_), brought on by comparatively small spin–orbit coupling (SOC) in the excited state and resulting in long excited-state lifetimes (∼10^−5^ s) that limit their prospects in OLED applications.

**Fig. 1:**
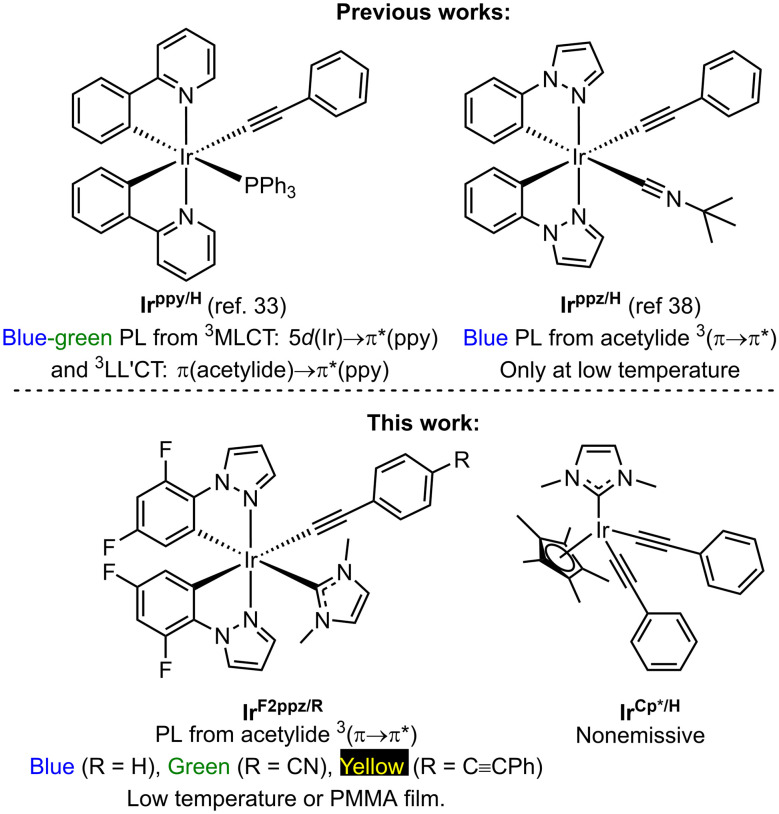
Research backgrounds on Ir(iii) acetylide complexes and our design.

A potential strategy to ameliorate this is to prepare Ir(iii) acetylide complexes where the emissive excited-state is acetylide-localized, since in many situations Ir(iii) engenders stronger SOC than Pt(ii) and thus could produce faster radiative rates and shorter lifetimes than Pt(ii).^31^ Nevertheless, this idea has been scarcely investigated. There are a few classes of cyclometalated Ir(iii) complexes containing acetylide ligands.^32–37^ In some of these, the cyclometalating ligand is 2-phenylpyridine (ppy), giving blue-to-green phosphorescence originating from a mixture of a ^3^MLCT state involving the ppy ligand and a triplet ligand-to-ligand charge transfer (^3^LL′CT) state involving the acetylide and ppy (Fig. 1).^33,37^ There is one isolated example, from a patent filed by Thompson and colleagues, showing that a bis-cyclometalated Ir(iii) complex containing phenylacetylide can elicit luminescence from a ^3^(π → π*) state localized on the acetylide ligand.^38^ This occurs when 1-phenyl-1*H*-pyrazole (ppz) is the cyclometalating ligand, which results in the ^3^MLCT energy being higher than that of the acetylide ^3^(π → π*) state (Fig. 1). However, phosphorescence only occurs at 77 K with a very long lifetime of 1460 µs.

In this work, we present an expanded investigation of Ir(iii) acetylide complexes, pursuing three key modifications of cyclometalated iridium complexes with acetylide ligands, to gain a deeper understanding of acetylide-centered luminescence in these compounds. First, we replace ppz with its fluorinated analogue 2-(2,4-difluorophenyl)pyrazole (F_2_ppz), which should destabilize the ^3^MLCT energy to an even greater extent, increasing the separation between the ^3^MLCT and acetylide ^3^(π → π*) states.^21,39^ In addition, we replace the isocyanide ancillary ligand used in Thompson's work^38^ with an NHC, a stronger σ-donor that should reduce thermal population of the ^3^MC state.^40,41^ Finally, we include two π-extended aryl acetylide analogues, which reduce the energy of the acetylide ^3^(π → π*) state and shift the PL to longer wavelengths.^29,42^ These investigations also made us ponder whether the cyclometalating ligands were needed at all in iridium(iii) compounds with acetylide-centered phosphorescence, so we also include an Ir(iii) bis(phenylacetylide) complex supported by 1,2,3,4,5-pentamethylcyclopentadienyl (Cp*) and the same NHC used in the cyclometalated complexes described here (Fig. 1). The organometallic chemistry of related Cp*Ir(iii) acetylide complexes has been investigated,^43–45^ but no photophysical studies of this structure type have been reported. There are some relevant reports of luminescent Cp*Ir(iii) complexes containing a bidentate chromophoric ligand, but none of these involve acetylide ligands.^46–49^ This work includes a detailed photophysical analysis of the four reported compounds, which shows that the cyclometalated complexes are weakly luminescent, but no luminescence is observed in the Cp* analogue.

## Results and discussion

### Synthesis of iridium(iii) complexes

**Scheme 1:**
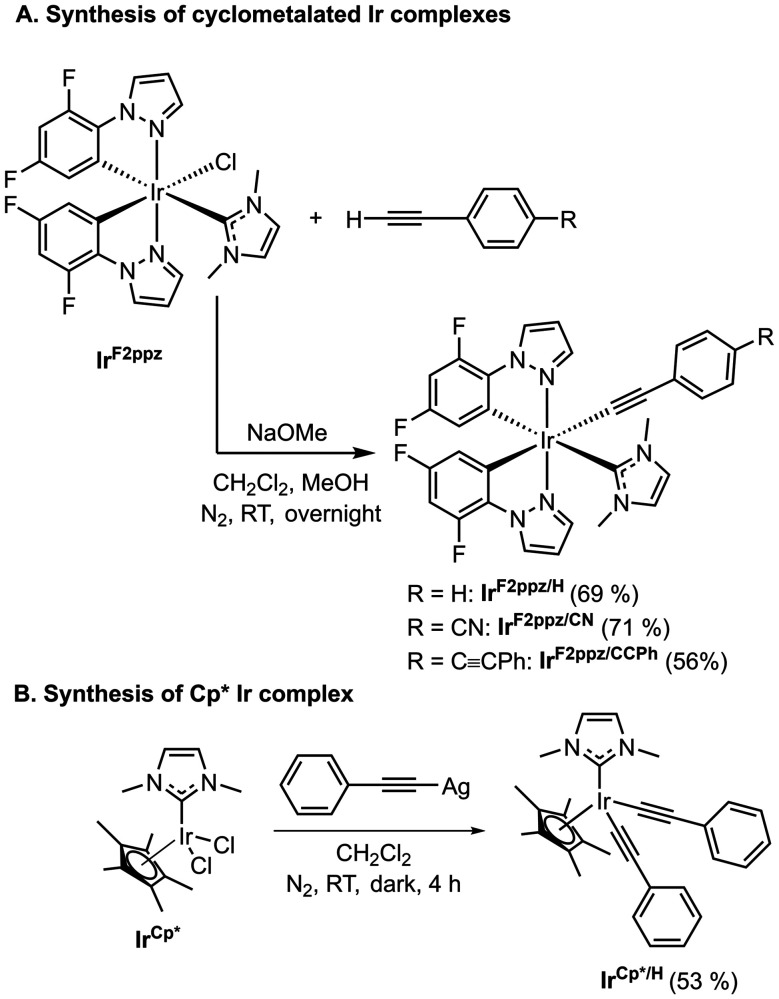
General schemes for the synthesis of Ir(iii) complexes.

The syntheses of the three cyclometalated Ir(iii) acetylide complexes of the general formula Ir(F_2_ppz)_2_(NHC)(C≡C-*p*-C_6_H_4_R) (F_2_ppz = 2-(2,4-difluorophenyl)pyrazole, NHC = 1,3-dimethylimidazol-2-ylidene, R = H, CN, or C≡CPh) are described in Scheme 1A. The compounds are abbreviated as Ir^F2ppz/R^, where R indicates the substituent at the 4-position of the substituted phenylacetylide ligand. The synthesis of the Cp* Ir(iii) acetylide complex, abbreviated as Ir^Cp*/H^, is outlined in Scheme 1B. The preparation of the chloride precursors (Ir^F2ppz^ and Ir^Cp*^) are described in the SI, following modified reported procedures.^27,50^ To prepare the cyclometalated complexes, Ir^F2ppz^ was treated with the respective arylacetylene in CH_2_Cl_2_ and methanol (MeOH) at room temperature under nitrogen, using sodium methoxide (NaOMe) as the base. An analogous approach to preparing Ir^Cp*/H^ was unsuccessful, so we instead accessed this complex by transmetallation between Ir^Cp*^ and (phenylethynyl)silver (PhCCAg) in CH_2_Cl_2_ in the dark.^51^ The four new acetylide complexes were purified by precipitation and isolated in moderate to good yields. All four reported Ir acetylide complexes are sensitive to ambient atmosphere and needed to be handled under inert atmosphere, inside a nitrogen-filled glove box. The structural identities and bulk purity of the products were confirmed by ^1^H NMR, ^13^C{^1^H} NMR, ^19^F NMR, and high-resolution mass spectrometry (Fig. S5–S12).

**Fig. 2:**
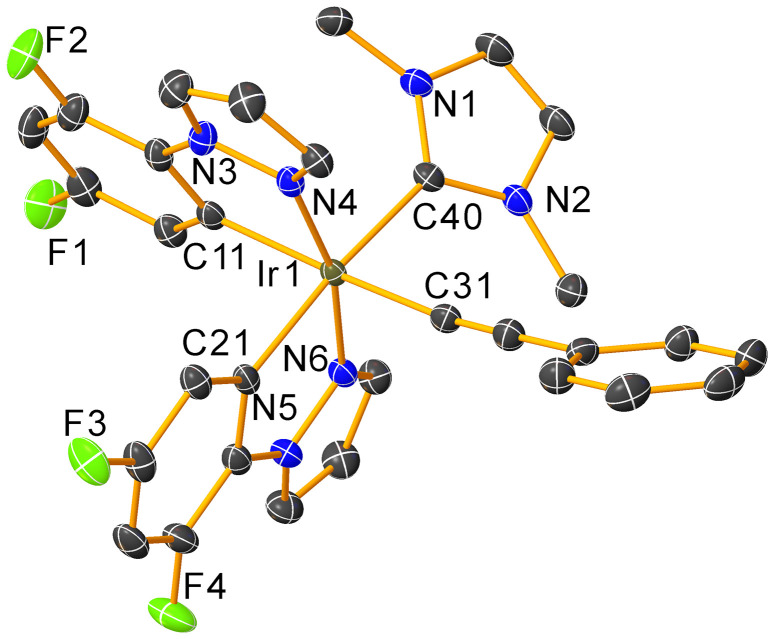
Molecular structure of Ir^F2ppz/H^, determined by single crystal X-ray diffraction. Thermal ellipsoids are drawn at the 50% probability level with the CH_2_Cl_2_ solvent molecule and hydrogen atoms eliminated. Selected bond distances (Å): Ir1–C11 = 2.061(2), Ir1–C21 = 2.059(8), Ir1–C31 = 2.057(5), Ir1–C40 = 2.115(3), Ir1–N4 = 2.021(2), Ir–N6 = 2.034(0). Bond angle (°): C11–Ir1–N4 = 80.01(7), N4–Ir1–C40 = 97.85(6), N4–Ir1–C31 = 89.71(7), C40–Ir1–C31 = 89.47(7).

The molecular structure of complex Ir^F2ppz/H^ was characterized by single-crystal X-ray diffraction and is displayed in Fig. 2. The detailed crystallographic data is given in Table S1 in the SI. Attempts to obtain X-ray quality single crystals of the remaining three complexes were not successful. In Ir^F2ppz/H^, a distorted octahedral geometry about the Ir center is observed. The two nitrogen atoms of the cyclometalating F_2_ppz ligands are in a *trans* disposition to one another, as typically observed for bis-cyclometalated iridium complexes, avoiding a *trans* arrangement of two strong *trans*-influencing cyclometalated aryl rings. The Ir–C(acetylide) bond distance observed in Ir^F2ppz/H^ is 2.0575(19) Å, which is not substantially different from other previously reported Ir–C(acetylide) bond lengths (within *ca.* 0.15 Å).^52,53^ The infrared (IR) spectra of all acetylide complexes are displayed in the SI, Fig. S13–16. The cyclometalated complexes Ir^F2ppz/H^, Ir^F2ppz/CN^, and Ir^F2ppz/CCPh^ each show a single C≡C stretching band at *ca.* 2080 cm^−1^. Meanwhile, two C≡C stretching bands were observed for Ir^Cp*/H^ at 2087 and 2048 cm^−1^, consistent with symmetric and asymmetric stretches arising from the two phenylacetylide ligands.

### Electrochemistry

**Fig. 3:**
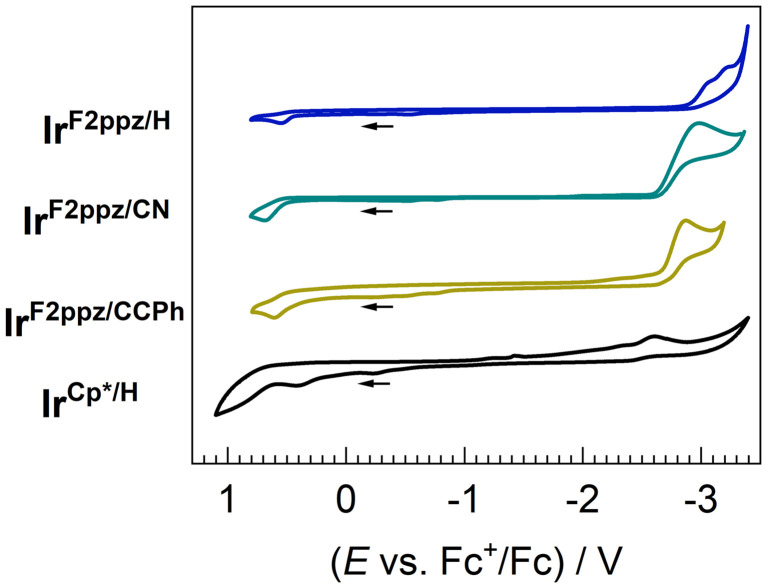
Overlaid cyclic voltammograms of Ir^F2ppz/H^, Ir^F2ppz/CN^, Ir^F2ppz/CCPh^, and Ir^Cp*/H^. The CVs were recorded at 0.1 V s^−1^ in acetonitrile with 0.1 M TBAPF_6_ as a supporting electrolyte. Potentials are referenced to an internal standard of ferrocene and currents are normalized.

The redox potentials of all four reported complexes were measured by cyclic voltammetry (CV), with their voltammograms shown in Fig. 3 and the electrochemical data summarized in Table 1. All redox waves of the Ir(iii) acetylide complexes are irreversible, which is also commonly observed in Pt(ii) acetylide complexes.^29,54^ Peak potentials are therefore reported in Table 1; the potential associated with electrochemical oxidation, the formal Ir^IV/III^ couple, is abbreviated as *E*^ox^, and the potential associated with one-electron reduction of the complexes is abbreviated as *E*^red^. The electrochemical HOMO–LUMO gaps (Δ*E*_H–L_) are estimated as the difference between these two potentials, *i.e.*, *E*^ox^ − *E*^red^. In the three cyclometalated complexes (Ir^F2ppz/H^, Ir^F2ppz/CN^, and Ir^F2ppz/CCPh^), varying the acetylide ligand results in changes in *E*^ox^, *E*^red^, and Δ*E*_H–L_, which suggests that the frontier orbitals of these complexes involve significant contributions from the acetylide ligand. Compared to complex Ir^F2ppz/H^ with the unsubstituted phenylacetylide, the cyano-substituted Ir^F2ppz/CN^ and π-extended Ir^F2ppz/CCPh^ showed anodic shifts (*i.e.*, more positive values) for both *E*^red^ and *E*^ox^, indicating stabilization of both HOMO and LUMO. This is also confirmed by DFT calculations (*vide infra*). Our group's previous work on varying the arylacetylide ligands in Pt(ii) complexes also showed that the introduction of electron-withdrawing groups on the acetylide stabilizes both frontier orbitals.^29^ Replacing the cyclometalating ligands by Cp* in Ir^Cp*/H^ destabilizes the HOMO and stabilizes the LUMO when compared to Ir^F2ppz/H^. A much smaller Δ*E*_H–L_ is also observed in Ir^Cp*/H^ than in the cyclometalated complexes reported here.

**Table 1:** Summary of electrochemical data^a^

Complex	*E* ^ox^/V	*E* ^red^/V	Δ*E*_H–L_ b/eV
Ir^F2ppz/H^	0.54	−3.06	3.60
Ir^F2ppz/CN^	0.68	−2.97	3.65
Ir^F2ppz/CCPh^	0.60	−2.86	3.46
Ir^Cp*/H^	0.40	−2.59	2.99

a Peak anodic (*E*_p,a_) and cathodic (*E*_p,c_) potentials are reported.

b Electrochemical HOMO–LUMO gap, estimated as the difference between the two redox potentials (*E*^ox^ − *E*^red^).

### Photophysical properties

**Fig. 4:**
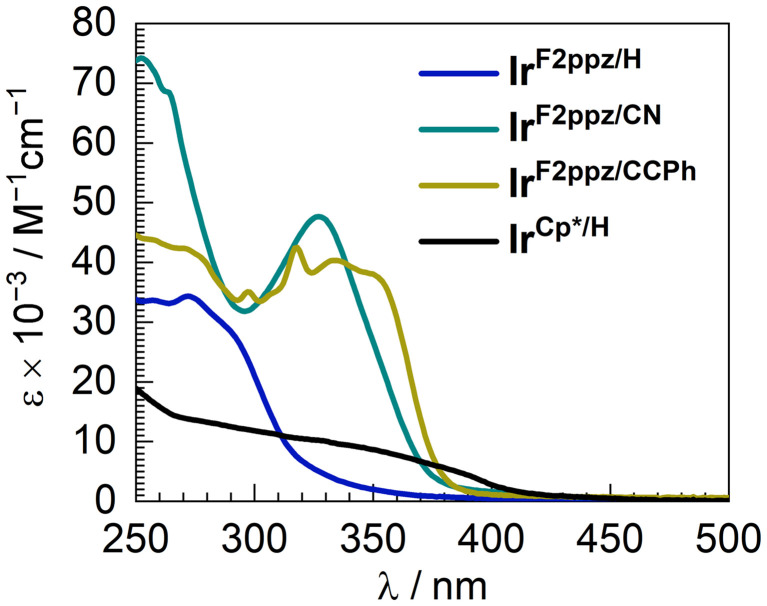
Overlaid UV-vis absorption spectra of four studied complexes, recorded in CH_2_Cl_2_ at room temperature.

Overlaid UV–vis absorption spectra of four reported complexes were recorded in CH_2_Cl_2_ at room temperature and are displayed in Fig. 4. Ir^F2ppz/H^, Ir^F2ppz/CN^, and Ir^F2ppz/CCPh^ show strong absorption bands in the UV region. These high-energy bands in all three cyclometalated complexes (250–300 nm) could be assigned to acetylide ligand-centered (LC) π → π* and ligand-to-ligand charge transfer (LL′CT) transitions involving the acetylide and F_2_ppz ligands, with some contributions from MLCT transitions, as suggested by DFT calculations (*vide infra*). In Ir^F2ppz/CN^ and Ir^F2ppz/CCPh^ there are additional bands in the region of 300–400 nm, arising from localized transitions involving the more conjugated arylacetylides. This assignment is corroborated by the more intense and longer-wavelength bands in the UV–vis absorption spectra of the substituted phenylacetylene derivatives, (4-CN)C_6_H_4_C≡CH and (4-PhC≡C)C_6_H_4_C≡CH, when compared to that of the unsubstituted phenylacetylene (Fig. S23, SI). The complex Ir^Cp*/H^, on the other hand, shows much weaker absorption than the three cyclometalated complexes with no clear maxima. The broad, monotonically decreasing absorption profile tails out to longer wavelengths than the rest, to nearly 450 nm.

**Fig. 5:**
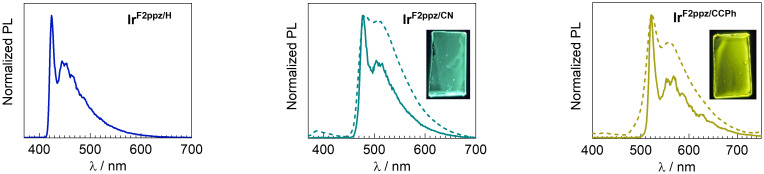
Overlaid photoluminescence spectra of Ir^F2ppz/H^, Ir^F2ppz/CN^, and Ir^F2ppz/CCPh^ at 77 K in CH_2_Cl_2_ (solid lines) and at room temperature in PMMA film, with 2 wt% of the iridium complex (dashed lines). The inset photographs show the doped PMMA films back-lit with a handheld UV lamp.

The three cyclometalated Ir complexes are not emissive in CH_2_Cl_2_ solution at room temperature. At 77 K, due to the suppression of nonradiative relaxation pathways at low temperatures, they show vibronically structured photoluminescence profiles with sharp 0–0 transitions (Fig. 5 and Table 2). The peak *λ*_0–0_ values progressively shift to longer wavelengths as the conjugation of the arylacetylide ligand increases (Ir^F2ppz/H^ < Ir^F2ppz/CN^ < Ir^F2ppz/CCPh^). With phenylacetylide in Ir^F2ppz/H^, blue phosphorescence is observed with *λ*_0–0_ = 424 nm, while the addition of an electron-withdrawing group and extending the arylacetylide conjugation in Ir^F2ppz/CN^ and Ir^F2ppz/CCPh^ give green (*λ*_0–0_ = 477 nm) and yellow (*λ*_0–0_ = 522 nm) PL, respectively. These bands are similar to those of previously reported Pt(ii) acetylide complexes with the same acetylide ligands.^29,42^ These characteristics make it clear that the PL in these three complexes arises from an acetylide-centered T_1_ excited state, ^3^(π → π*), as also suggested by the DFT calculations described below. This is further corroborated by the similarity between the 77 K PL spectra of Ir^F2ppz/H^ and Ir^F2ppz/CN^ and those of their free acetylene ligands, which exhibit weak phosphorescence at low temperature (Fig. S24, SI). In PMMA film at room temperature, doped with 2 wt% of the respective complex, only Ir^F2ppz/CN^ and Ir^F2ppz/CCPh^ luminesce, with peak wavelengths that are very similar to those observed at 77 K in solution, albeit with vibronic profiles that are less resolved. Both complexes luminesce weakly in PMMA film, with quantum yields and lifetimes that are below the limits of accurate measurement on our instruments. The excitation spectra of these two complexes were collected in PMMA film and are well overlaid with their solution UV–vis absorption spectra (Fig. S21 and S22), indicating that the luminescence can be confidently assigned to the complex without interference of minor impurities. PL spectra of the three cyclometalated complexes recorded at different excitation wavelengths are displayed in Fig. S25–S27 of the SI. There are minimal changes, indicating no dependence of the observed emission on the excitation wavelength. Complex Ir^Cp*/H^, on the other hand, is not luminescent in any of the media we examined (solution at room temperature and 77 K, or PMMA film at room temperature).

**Table 2:** Summary of photoluminescence data of the cyclometalated iridium complexes Ir^F2ppz/R^

Complex	PL in CH_2_Cl_2_ at 77 K	PL in PMMA film at room temperature, 2 wt%	
	*λ*/nm	*λ*/nm	*Φ*_PL_
Ir^F2ppz/H^	424, 445, 453	a	a
Ir^F2ppz/CN^	477, 504	483, 508	<0.01
Ir^F2ppz/CCPh^	522, 553, 568	524, 560	<0.01

a Not emissive.

In typical bis-cyclometalated iridium acetylide complexes, phosphorescence arises from a mixture of a ^3^MLCT state involving the conjugated cyclometalating C^^^N ligands and a ^3^LL′CT state involving acetylide and C^^^N ligands. Cyclometalated Ir(iii) acetylide complexes with ppy as the C^^^N ligand exhibit this paradigm, where the ^3^MLCT state is lower in energy than the acetylide-centered ^3^(π → π*) state. Work from Fu *et al.* showed that complexes of this type are phosphorescent in room-temperature solutions with good PL quantum yields.^33^ In contrast, our goal in this work was to access iridium(iii) acetylide complexes where phosphorescence originates from a ^3^(π → π*) state localized on the acetylide. This possibility was realized in the previously reported complex Ir^ppz/H^ (Fig. 1), reported by Thompson and colleagues, which only luminesces at 77 K.^38^ In this work, we sought two modifications that we hypothesized could improve the photophysical properties. First, replacing ppz with the fluorinated analogue F_2_ppz should increase the energetic separation between the ^3^MLCT state (involving the cyclometalating ligand) and the acetylide-centered ^3^(π → π*) state. In addition, we reasoned that the *tert*-butyl isocyanide ligand in Ir^ppz/H^ may not be effective at destabilizing ^3^MC states and preventing their thermal population, motivating the switch to the NHC ligand in Ir^F2ppz/H^. However, these modifications did not noticeably improve the PL in Ir^F2ppz/H^, which analogous to Ir^ppz/H^ only exhibits its deep-blue luminescence when cooled to 77 K. We did observe room-temperature phosphorescence in the more conjugated analogues Ir^F2ppz/CN^ and Ir^F2ppz/CCPh^, which luminesce weakly in the green and yellow regions at room temperature, when immobilized in PMMA films. It is interesting to note that many reported Pt(ii) arylacetylide complexes give appreciable photoluminescence quantum yields in the blue region and beyond, from acetylide-centered ^3^(π → π*) states.^42,55–58^ Complex Ir^Cp*/H^ is not luminescent at either room temperature or 77 K. Photophysical studies on related complexes have been carried out, on compounds of the general formula [Cp*Ir(C^N)(L)]^*n*+^ (C^N = cyclometalating ligand) and [Cp*Ir(bpy)(L)]^*m*+^, where L is a variable strong σ-donating ancillary ligand and bpy is 2,2'-bipyridine.^46^ The authors of this prior work observed low-energy ligand-to-ligand charge transfer (LL′CT) excited states in some of the complexes. Although the relationship of these previous examples to Ir^Cp*/H^ may be specious since the acetylide complex reported here lacks a conjugated chelating C^N or bpy ligand, we nonetheless speculate that similar low-energy charge-transfer states may be responsible for quenching the desired acetylide-centered ^3^(π → π*) phosphorescence in Ir^Cp*/H^. Nevertheless, the outcomes of this work show that although acetylide-centered ^3^(π → π*) phosphorescence in iridium complexes can be engineered by the appropriate combination of cyclometalating and acetylide ligands, it is not a design strategy that is likely to produce compounds with highly desirable photophysical properties.

### DFT calculations

**Fig. 6:**
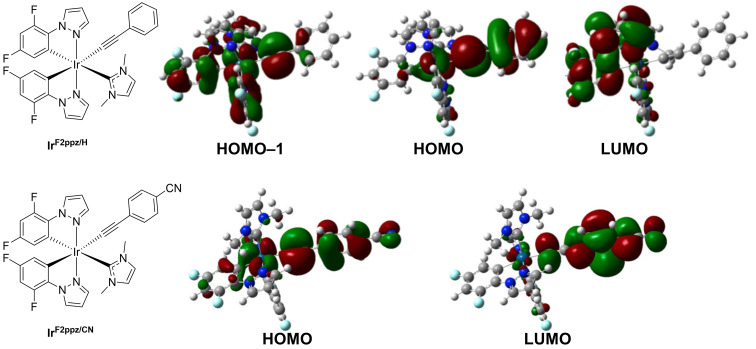
Main molecular orbitals involved in S_0_ → S_1_ excitations of Ir^F2ppz/H^ and Ir^F2ppz/CN^.

Time-dependent density functional theory (TD-DFT) calculations were carried out for Ir^F2ppz/H^ and Ir^F2ppz/CN^ to characterize the S_0_ → S_1_ and S_0_ → T_1_ transitions. A summary of the calculated HOMO and LUMO energies with SMD(CH_2_Cl_2_) solvation is shown in Table 3, and the corresponding values computed in the gas phase are shown in Table S2 in the SI. In agreement with the CV data, the cyano group in Ir^F2ppz/CN^ stabilizes both the HOMO and LUMO energies relative to Ir^F2ppz/H^. A smaller HOMO–LUMO gap also is observed for Ir^F2ppz/CN^. Frontier molecular orbitals involved in the S_0_ → S_1_ transitions are shown in Fig. 6.

**Table 3:** Calculated HOMO and LUMO energies for complexes Ir^F2ppz/H^ and Ir^F2ppz/CN^, computed with SMD(CH_2_Cl_2_) solvation

Compound	HOMO	LUMO	HOMO–LUMO gap
	*E*/eV	*E*/eV	*E*/eV (*λ*/nm)
Ir^F2ppz/H^	−0.246	4.211	4.457 (278)
Ir^F2ppz/CN^	−0.252	3.747	3.999 (310)

In Ir^F2ppz/H^, the S_0_ → S_1_ excitation (278 nm, oscillator strength, *f* = 0.1207) has two major transitions: HOMO → LUMO (35%) and HOMO−1 → LUMO (42%). As shown in Fig. 6 (top), the HOMO and HOMO−1 orbitals of Ir^F2ppz/H^ are dπ* orbitals; the HOMO involves substantial contribution from the phenylacetylide π system and the HOMO−1 is a more delocalized orbital that involves the second perpendicular C≡C π orbital and the cyclometalating ligands. The LUMO primarily involves the cyclometalating ligand F_2_ppz. Thus, the longest-wavelength UV-vis absorption band in Ir^F2ppz/H^ can be assigned to a mixture of acetylide → F_2_ppz LL'CT and Ir → F_2_ppz MLCT transitions.

In Ir^F2ppz/CN^, the S_0_ → S_1_ excitation primarily involves a HOMO → LUMO transition (81%), with a longer peak wavelength (310 nm) and a much higher oscillator strength (*f* = 1.246). The HOMO in the cyano-substituted analogue also is a dπ* orbital with delocalization onto the arylacetylide, but the LUMO is quite different and is almost exclusively an acetylide π* orbital. This arrangement gives rise to an intense S_0_ → S_1_ excitation and supports the assignment of the low-energy absorption band in Ir^F2ppz/CN^ as a localized transition of the 4-cyanophenylacetylide ligand. The computed UV-vis absorption spectra for Ir^F2ppz/H^ and Ir^F2ppz/CN^ are displayed in Fig. S28 and S29 of the SI, showing similar profiles as the experimental spectra albeit with a systematic blue shift.

TD-DFT calculations for the S_0_ → T_1_ transitions of Ir^F2ppz/H^ and Ir^F2ppz/CN^ show that the orbitals involved are localized primarily on the acetylide ligands, as depicted in Fig. S30 and S31. For Ir^F2ppz/H^, the T_1_ state at 3.04 eV is dominated by the HOMO → LUMO+3 transition (69.94%). For Ir^F2ppz/CN^, the T_1_ state at 2.70 eV is characterized by the HOMO → LUMO transition (71.17%). These results confirm the experimental conclusion that the phosphorescence in each case originates from a T_1_ state localized on the acetylide ligand.

## Conclusions

In this work, we describe four new Ir(iii) acetylide complexes, including three cyclometalated complexes and one Cp* complex that all contain NHCs as ancillary ligands. The cyclometalated Ir(iii) complexes are not emissive in CH_2_Cl_2_ solution at room temperature but luminesce at 77 K, with the emission profile dependent on the identity of the acetylide ligand. The complex with phenylacetylide emits in the blue region but, despite being supported by the NHC as a strong-field ancillary ligand, is not emissive at room temperature in both solution and PMMA film. The two other complexes are also very weakly emissive in PMMA film at room temperature. The Cp* complex is not luminescent. In addition to their poor photoluminescence efficiency, the compounds presented here are unstable in ambient atmosphere, foreshadowing bleak prospects for these compounds in optoelectronic applications. Nonetheless, this work presents a synthetic advance in being able to access complexes of this type, with the fundamental insight that acetylide-centered phosphorescence can be accessed in Ir(iii) complexes with judicious choice of supporting ligands.

## Experimental section

### Materials

Commercially available reagents were used without purification unless otherwise noted. Solvents for optical measurements were dried and deoxygenated using a Grubbs solvent purification pressurized with argon. The syntheses of chloride precursors Ir^F2ppz^ and Ir^Cp*^ are described in the SI (page S3). (Phenylethynyl)silver (PhCCAg) was prepared following a reported procedure in a one-step synthesis.^59^

### Physical methods

^1^H, ^13^C{^1^H}, and ^19^F NMR spectra were recorded at room temperature using a JEOL ECA-500 spectrometer. The ESI-MS experiments (Fig. S17–S20) were carried out at The University of Texas at Austin's Mass Spectrometry Facility on an Agilent Technologies 6530 accurate-mass Q-TOF LC/MS instrument. UV–vis absorption spectra were measured in CH_2_Cl_2_ in screw-capped 1 cm quartz cuvettes using an Agilent Cary 8454 UV–vis spectrophotometer. Photoluminescence (PL) spectra were collected using a Horiba FluoroMax-4 spectrofluorometer with a 370 nm long-pass filter to exclude the stray excitation light from detection. Samples for PL spectra were prepared in a nitrogen-filled glovebox using solvents obtained from the Grubbs solvent purification system. For PL measurements at 77 K, the sample was contained in a custom quartz EPR tube with a high-vacuum valve and cooled in liquid nitrogen using a quartz dewar sample holder specifically designed for the fluorimeter's sample chamber. Thin-film poly(methylmethacrylate) (PMMA) samples were prepared inside the nitrogen-filled glovebox at room temperature by drop-coating a quartz slide with a solution of PMMA (98 mg) and respective iridium complex (2.0 mg) dissolved in 1 mL of CH_2_Cl_2_. The absolute quantum yields of complexes doped into PMMA films were measured by using a Spectralon-coated integrating sphere integrated with a Horiba FluoroMax-4 spectrofluorometer. Cyclic voltammetry measurements were conducted with a CH Instrument 602E potentiostat using a three-electrode system, interfaced with a nitrogen glovebox *via* wire feedthroughs. Measurements were carried out in acetonitrile solution with 0.1 M TBAPF_6_ as a supporting electrolyte, by using a 3 mm diameter glassy carbon working electrode, Pt wire counter electrode, and silver wire pseudoreference electrode. All reported potentials were referenced to an internal standard of ferrocene. Infrared (IR) spectra were obtained on neat powders using a Thermo Nicolet Avatar FT-IR spectrometer with a diamond ATR.

### X-ray crystallography details

A single crystal of Ir^F2ppz/H^ was mounted on a Bruker Apex II three-circle diffractometer using Mo Kα radiation (*λ* = 0.71073 Å). The data was collected at 123(2) K, then processed and refined within the APEXII software. Structures were solved by intrinsic phasing in SHELXT and refined by standard difference Fourier techniques in the program SHELXL.^60^ Hydrogen atoms were placed in calculated positions using the standard riding model and refined isotropically; all non-hydrogen atoms anisotropically. Crystallographic details are summarized in Table S1.

### Computational details

Geometry optimizations for Ir^F2ppz/H^ and Ir^F2ppz/CN^ were carried out at ωB97X-D/def2-TZVPP and vibrational frequency analyses confirmed the nature of the minima structures. HOMO–LUMO gaps, vertical excitation energies, as well as the S_0_ → S_1_ and S_0_ → T_1_ transitions were evaluated at the TD-CAM-B3LYP/def2-TZVPP//ωB97X-D/def2-TZVPP level. TD-DFT calculations were performed on optimized S_0_ geometries and included 50 excited states. Singlet excited states were computed with implicit CH_2_Cl_2_ solvation employing the SMD model, whereas S_0_ → T_1_ transitions were evaluated in the gas phase using the same number of excited states. All calculations were performed with Gaussian 16 (Revision C.01).^61^

### General procedure for the synthesis of Ir^F2ppz/R^

Inside a glovebox, a 20 mL vial equipped with a magnetic stir bar was charged with the respective arylacetylene (2.0 equiv., 0.2 mmol), NaOMe (3.0 equiv., 0.30 mmol, 16 mg), and 3 mL of methanol. This mixture was stirred for 15 min and was then slowly added to another 20 mL vial containing Ir^F2ppz^ (1.0 equiv., 0.10 mmol, 68 mg) dissolved in 3 mL of CH_2_Cl_2_. The reaction mixture was stirred overnight and then concentrated to dryness under vacuum. After that, the residue was redissolved in CH_2_Cl_2_ and filtered. The filtrate was dried under vacuum and redissolved in a small amount of CH_2_Cl_2_ (1–2 mL), followed by an addition of 10 mL of pentane to precipitate the desired product out of the mixture, which was then collected by filtration. The product was washed with pentane and dried under vacuum.

Ir^F2ppz/H^. Prepared by the general procedure using phenylacetylene (0.20 mmol, 20 mg). Yield: 51 mg (69%) of a white solid. ^1^H NMR (500 MHz, CD_3_CN) *δ* 8.46 (d, *J* = 3.0 Hz, 1H, Ar*H*), 8.40 (d, *J* = 3.0 Hz, 1H, Ar*H*), 8.32 (d, *J* = 2.2 Hz, 1H, Ar*H*), 7.61 (d, *J* = 2.5 Hz, 1H, Ar*H*), 7.09 (t, *J* = 7.6 Hz, 2H, Ar*H*), 7.06–6.94 (m, 4H, Ar*H*), 6.83 (d, *J* = 2.1 Hz, 1H, Ar*H*), 6.75 (t, *J* = 2.5 Hz, 1H, Ar*H*), 6.68 (t, *J* = 2.6 Hz, 1H, Ar*H*), 6.50 (ddd, *J* = 12.2, 7.7, 2.9 Hz, 2H, Ar*H*), 5.73 (dd, *J* = 8.1, 2.4 Hz, 1H, Ar*H*), 5.47–5.40 (m, 1H, Ar*H*), 4.17 (s, 3H, NC*H*_3_), 2.63 (s, 3H, NC*H*_3_). ^19^F NMR (470 MHz, CD_3_CN) *δ* −116.35 (td, *J* = 8.6, 4.8 Hz, 1F), −116.49 (td, *J* = 8.5, 4.9 Hz, 1F), −126.22 (dd, *J* = 12.4, 5.0 Hz, 1F), −126.91 (dd, *J* = 12.4, 4.6 Hz, 1F). FT-IR: *ν̃*(C≡C) = 2087 cm^−1^. HRMS-SI: (*m*/*z*): [M + H]^+^ calcd for C_31_H_23_F_4_IrN_6_, 749.1624; found, 749.1602.

Ir^F2ppz/CN^. Prepared by the general procedure using 4-ethynylbenzonitrile (0.20 mmol, 25 mg). Yield: 54 mg (71%) of a light-yellow solid. ^1^H NMR (500 MHz, CD_3_CN) *δ* 8.46 (d, *J* = 3.0 Hz, 1H, Ar*H*), 8.39 (d, *J* = 3.0 Hz, 1H, Ar*H*), 8.27 (s, 1H, Ar*H*), 7.61 (s, 1H, Ar*H*), 7.41 (d, *J* = 8.1 Hz, 2H, Ar*H*), 7.09 (d, *J* = 8.0 Hz, 2H, Ar*H*), 7.04 (s, 1H, Ar*H*), 6.82 (s, 1H, Ar*H*), 6.74 (t, *J* = 2.5 Hz, 1H, Ar*H*), 6.68 (t, *J* = 2.5 Hz, 1H, Ar*H*), 6.54–6.46 (m, 2H, Ar*H*), 5.72 (dd, *J* = 8.2, 2.5 Hz, 1H, Ar*H*), 5.44 (dd, *J* = 8.2, 2.5 Hz, 1H, Ar*H*), 4.12 (s, 3H, NC*H*_3_), 2.62 (s, 3H, NC*H*_3_). ^19^F NMR (470 MHz, CD_3_CN) *δ* −116.14 to −116.24 (m, 1F), −116.30 to −116.40 (m, 1F), −126.11 (dd, *J* = 12.3, 5.0 Hz, 1F), −126.76 (dd, *J* = 12.3, 4.7 Hz, 1F). FT-IR: *ν̃*(C≡C) = 2083 cm^−1^, *ν̃*(C≡N) = 2218 cm^−1^. HRMS-SI: (*m*/*z*): [M + Na]^+^ calcd for C_32_H_22_F_4_IrN_7_, 796.1396; found, 796.1384. The ^19^F NMR spectrum shows a *ca.* 10% impurity that appears to be non-luminescent based on the well-overlaid absorption and excitation spectra.

Ir^F2ppz/CCPh^. Prepared by the general procedure using 1-ethynyl-4-(phenylethynyl)benzene (0.20 mmol, 40 mg). Yield: 47 mg (56%) of a yellow solid. ^1^H NMR (500 MHz, CD_3_CN) *δ* 8.46 (d, *J* = 2.9 Hz, 1H, Ar*H*), 8.40 (d, *J* = 3.0 Hz, 1H, Ar*H*), 8.31 (d, *J* = 2.3 Hz, 1H, Ar*H*), 7.62 (d, *J* = 2.4 Hz, 1H, Ar*H*), 7.49–7.43 (m, 2H, Ar*H*), 7.37–7.32 (m, 3H, Ar*H*), 7.25 (d, *J* = 7.9 Hz, 2H, Ar*H*), 7.06–6.98 (m, 3H, Ar*H*), 6.83 (d, *J* = 2.0 Hz, 1H, Ar*H*), 6.75 (t, *J* = 2.5 Hz, 1H, Ar*H*), 6.69 (t, *J* = 2.5 Hz, 1H, Ar*H*), 6.51 (ddd, *J* = 11.7, 9.0, 2.6 Hz, 2H, Ar*H*), 5.74 (dd, *J* = 8.0, 2.4 Hz, 1H, Ar*H*), 5.45 (dd, *J* = 8.1, 2.4 Hz, 1H, Ar*H*), 4.15 (s, 3H, NC*H*_3_), 2.63 (s, 3H, NC*H*_3_). ^19^F NMR (470 MHz, CD_3_CN) *δ* −116.24 (td, *J* = 8.6, 4.5 Hz, 1F), −116.40 (td, *J* = 8.5, 4.9 Hz, 1F), −126.15 (dd, *J* = 12.3, 4.9 Hz, 1F), −126.81 (dd, *J* = 12.3, 4.6 Hz, 1F). FT-IR: *ν̃*(C≡C) = 2083 cm^−1^. HRMS-SI: (*m*/*z*): [M + H]^+^ calcd for C_39_H_27_F_4_IrN_6_, 849.1937; found, 849.1921.

### Procedure for the synthesis of Ir^Cp*/H^

Inside a glovebox, a 20 mL vial equipped with a magnetic stir bar was charged with Ir^Cp*^ (1.0 equiv., 0.10 mmol, 49 mg), silver(i) phenylacetylide (PhC≡CAg, 2.0 equiv., 0.20 mmol, 42 mg), and 6 mL of CH_2_Cl_2_. The mixture was stirred in the dark for 4 h and then concentrated to dryness under vacuum. The crude product was redissolved in a small amount of CH_2_Cl_2_ (1–2 mL), followed by an addition of 10 mL of pentane to precipitate the desired product from the mixture, which was then collected by filtration. The product was washed with pentane and dried under vacuum. Yield: 29 mg (53%) of a reddish solid. ^1^H NMR (500 MHz, CDCl_3_) *δ* 7.34–7.31 (m, 4H, Ar*H*), 7.13 (t, *J* = 7.7 Hz, 4H, Ar*H*), 7.01–6.97 (m, 2H, Ar*H*), 6.85 (s, 2H, Ar*H*), 3.97 (s, 6H, NC*H*_3_), 1.90 (s, 15H, CC*H*_3_). ^13^C{^1^H} NMR (101 MHz, CDCl_3_) *δ* 148.3, 132.0, 130.3, 127.5, 123.9, 122.7, 100.0, 93.3, 82.9, 38.9, 9.5. FT-IR: *ν̃*(C≡C) = 2087, 2048 cm^−1^. HRMS-SI: (*m*/*z*): [M + H]^+^ calcd for C_31_H_33_IrN_2_, 627.2347; found, 627.2358.

## Author contributions

Son N. T. Phan: formal analysis, investigation, validation, visualization, writing – original draft, writing – review & editing. João V. Schober: formal analysis, investigation, visualization, writing – review & editing. Judy I. Wu: funding acquisition, supervision, writing – review & editing. Thomas S. Teets: conceptualization, formal analysis, funding acquisition, project administration, supervision, visualization, writing – review & editing.

## Conflicts of interest

There are no conflicts to declare.

## Supplementary Material

DT-055-D5DT02734A-s001

DT-055-D5DT02734A-s002

## Data Availability

The data supporting this article have been included as part of the supplementary information (SI). Supplementary information: X-ray crystallography summary tables, NMR spectra, ESI-MS data, additional photophysical data, data from DFT calculations. See DOI: https://doi.org/10.1039/d5dt02734a. CCDC 2489655 (Ir^F2ppz/H^) contains the supplementary crystallographic data for this paper.^62^
