# Supplementary material for: Synthesis and photoluminescence of iridium(iii) arylacetylide complexes with acetylide-localized emissive excited states
Source: Dalton Trans. 2026 Jan 27;55(6):2499–508. doi: 10.1039/d5dt02734a (PMC12836165; doi:10.1039/d5dt02734a)
Supplement: DT-055-D5DT02734A-s001 [file DT-055-D5DT02734A-s001.pdf]

Supplementary Information (SI) for Dalton Transactions.
This journal is © The Royal Society of Chemistry 2026

Supplementary Information

Synthesis and photoluminescence of iridium(III) arylacetylide complexes with acetylide-localized emissive excited states

Son N. T. Phan, João V. Schober, Judy I. Wu, and Thomas S. Teets*

University of Houston, Department of Chemistry 3585 Cullen Blvd., Room 112, Houston, Texas, 77204-5003, United States.

*Corresponding author: tteets@uh.edu

Index	*Page*
X-ray crystallographic summary table	S2
Additional synthetic details	S3
NMR spectra of complexes	S4–S9
IR spectra of complexes	S10–S11
ESI-MS accurate mass reports of complexes	S12–S13
Overlaid UV–vis absorption and excitation spectra	S14
UV–vis absorption spectra of free ligands	S15
77 K PL spectra of phenylacetylene and 4-cyanophenylacetylene	S15
Excitation-wavelength dependent PL spectra of cyclometalated complexes	S16–S17
Computed HOMO and LUMO energies in the gas phase	S18
Computed UV–vis absorption spectra for $\mathbf{Ir^{F2ppz/H}}$ and $\mathbf{Ir^{F2ppz/CN}}$	S18–S19
Orbitals involved in $S_0{\rightarrow}T_1$ transition of $\mathbf{Ir^{F2ppz/H}}$ and $\mathbf{Ir^{F2ppz/CN}}$	S19
Cartesian coordinates of the optimized geometries from DFT calculations	S20–S24
References	S25

Table S1. Summary of X-ray crystallographic data for the complex **Ir$^{F2ppz/H}$**·CH_2Cl_2.

	**Ir$^{F2ppz/H}$**·CH_2Cl_2
CCDC	2489655
**Crystal data**	
Chemical formula	$C_{32}H_{25}Cl_2F_4IrN_6$
M_r	832.68
Crystal system, space group	Triclinic, $P\bar{1}$
Temperature (K)	150
a, *b*, *c* (Å)	10.9423 (13), 11.8086 (14), 13.0470 (16)
α, β, γ (°)	111.781 (1), 90.373 (1), 97.365 (1)
V (Å^3)	1549.9 (3)
Z	2
Radiation type	Mo $K\alpha$
μ (mm^{-1})	4.54
Crystal size (mm)	0.28 × 0.25 × 0.14
**Data collection**	
Diffractometer	Bruker *APEX*-II CCD
Absorption correction	Multi-scan<br>*SADABS*
T_{min}, T_{max}	0.587, 0.746
No. of measured, independent and observed [$I > 2\sigma(I)$] reflections	21752, 6800, 6582
R_{int}	0.015
$(\sin \theta/\lambda)_{max}$ (Å^{-1})	0.641
**Refinement**	
$R[F^2 > 2\sigma(F^2)]$, $wR(F^2)$, S	0.014, 0.034, 1.05
No. of reflections	6800
No. of parameters	408
H-atom treatment	H-atom parameters constrained
$\Delta\rho_{max}$, $\Delta\rho_{min}$ (e Å^{-3})	0.92, −0.64

Additional synthetic details

Synthesis of $\mathbf{Ir^{F2ppz}}$: Synthesized followed a modified procedure.[1] To a 50-mL round bottom flask equipped with a magnetic stir bar was added **[Ir(F$_2$ppz)$_2$(μ-Cl)]$_2$** (0.101 mmol, 118 mg, prepared following a reported procedure[2]), Ag_2O (0.12 mmol, 28 mg), 1,3-dimethyl-1*H*-imidazol-3-ium iodide (0.24 mmol, 54 mg), and 1,2-dichloroethane (12 mL). The reaction mixture was refluxed at 80 °C overnight. Upon completion, the mixture was filtered, and the filtrate was collected and concentrated and then subjected to column chromatography on silica gel (ethyl acetate/dichloromethane = 1:4.5 v/v) to obtain the desired product as a white solid. Yield: 103 mg (76%). ^{1}H NMR (500 MHz, $CDCl_3$) δ 8.37 (d, *J* = 2.9 Hz, 1H, Ar*H*), 8.34 (d, *J* = 2.5 Hz, 2H, Ar*H*), 7.61 (d, *J* = 2.3 Hz, 1H, Ar*H*), 6.85 (d, *J* = 1.9 Hz, 1H, Ar*H*), 6.73 (t, *J* = 2.6 Hz, 1H, Ar*H*), 6.66 (d, *J* = 1.8 Hz, 1H, Ar*H*), 6.58 (t, *J* = 2.6 Hz, 1H, Ar*H*), 6.44–6.35 (m, 2H, Ar*H*), 5.67 (ddd, *J* = 8.8, 2.5, 0.9 Hz, 1H), 5.43 (dd, *J* = 8.0, 2.5 Hz, 1H, Ar*H*), 4.16 (s, 3H, CH_3), 2.79 (s, 3H, CH_3). ^{19}F NMR (470 MHz, $CDCl_3$) δ −113.41 (td, *J* = 8.3, 5.4 Hz, 1F), −114.15 (td, *J* = 8.7, 5.2 Hz, 1F), −125.09 (dd, *J* = 12.0, 5.3 Hz, 1F), −125.58 (dd, *J* = 12.3, 5.2 Hz, 1F).

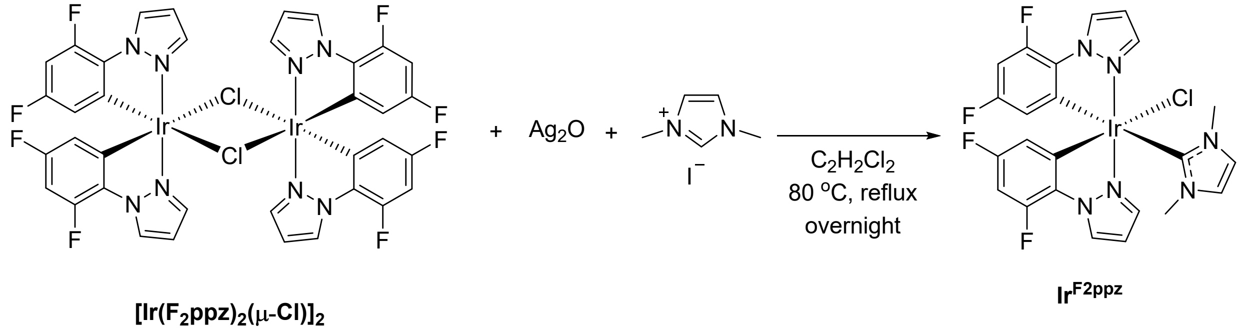

Scheme S1. Synthesis of $\mathbf{Ir^{F2ppz}}$.

Synthesis of $\mathbf{Ir^{Cp*}}$: Synthesized followed a modified procedure.[3] To a 20-mL vial equipped with a magnetic stir bar was added **[Ir(Cp*)(μ-Cl)]$_2$** (0.050 mmol, 40 mg), **AgNHC** (0.050 mmol, 33 mg, prepared following a reported procedure[4]), and CH_2Cl_2 (5 mL). The mixture was stirred at room temperature for 1 h. Upon completion, the mixture was filtered to remove the precipitate. The filtrate was dried under vacuum to remove the solvent and the crude product redissolved in 1 mL of CH_2Cl_2, followed by an addition of 10 mL of hexane to precipitate the product out of the mixture, which was then collected by filtration. The product was washed with hexane. Orange solid. Yield: 58 mg (93%).^{1}H NMR (500 MHz, $CDCl_3$) δ 6.90 (s, 2H, Ar*H*), 3.93 (s, 6H, NCH_3), 1.60 (s, 15H, CCH_3). ^{13}C{^{1}H} NMR (126 MHz, $CDCl_3$) δ 156.3, 123.3, 88.7, 38.7, 9.3.

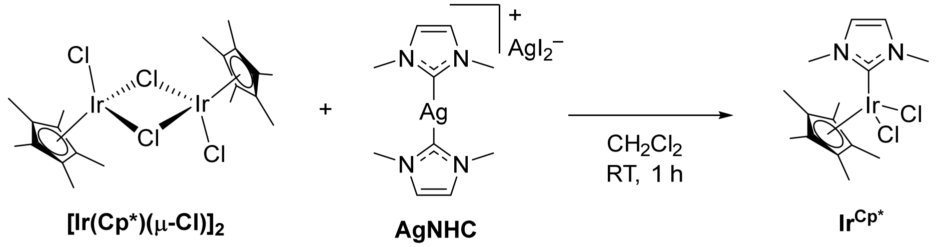

Scheme S2. Synthesis of $\mathbf{Ir^{Cp*}}$.

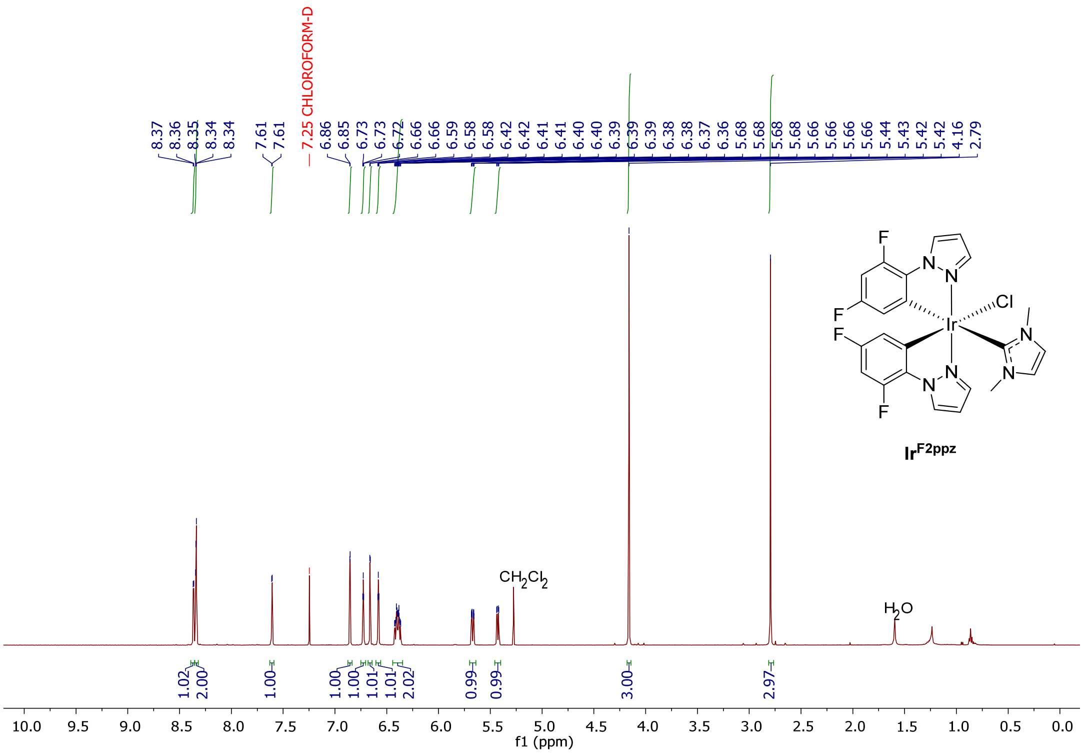

Fig. S1. ^{1}H NMR spectrum of complex **Ir^{F2ppz}**, recorded in chloroform-*d* at 500 MHz.

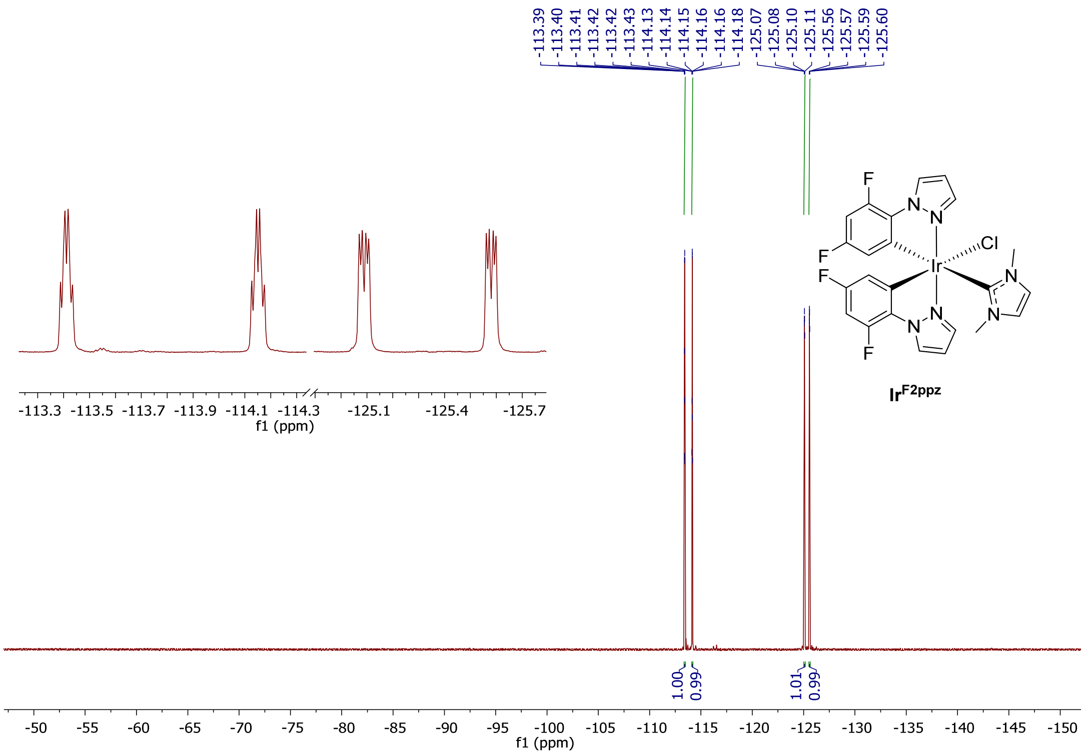

Fig. S2. ^{19}F NMR spectrum of complex **Ir^{F2ppz}**, recorded in chloroform-*d* at 470 MHz.

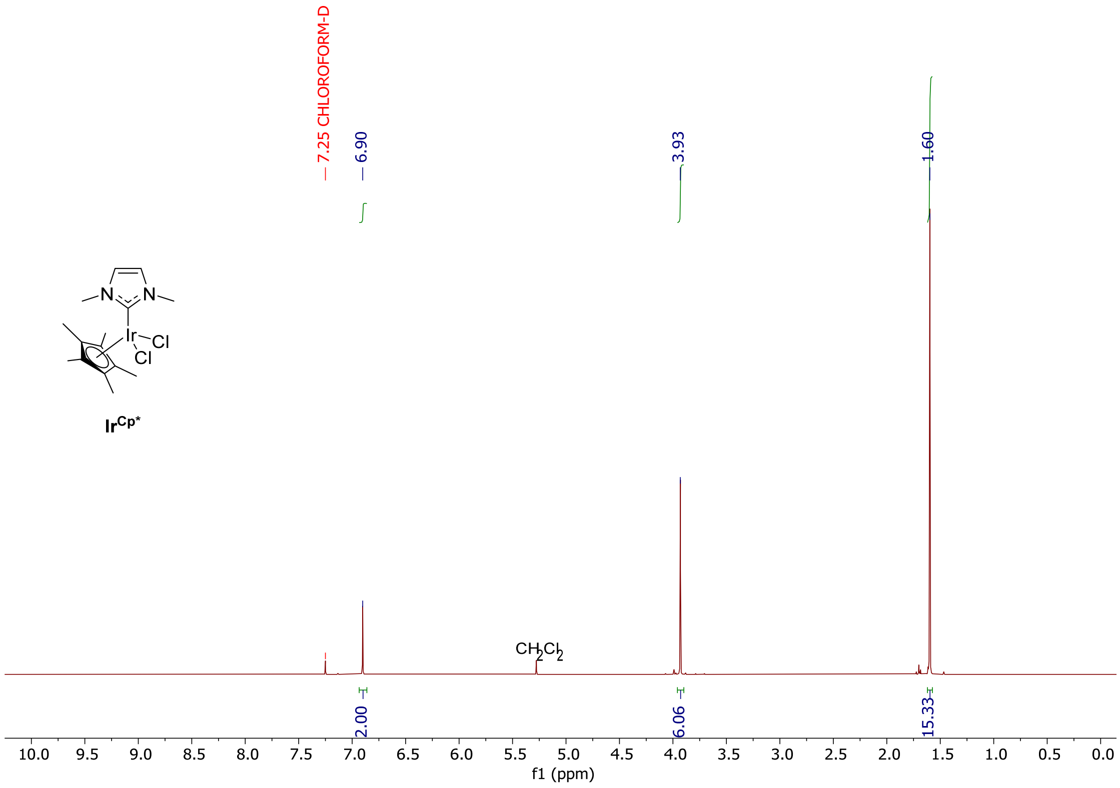

Fig. S3. ^{1}H NMR spectrum of complex **Ir^{Cp*}**, recorded in chloroform-*d* at 500 MHz.

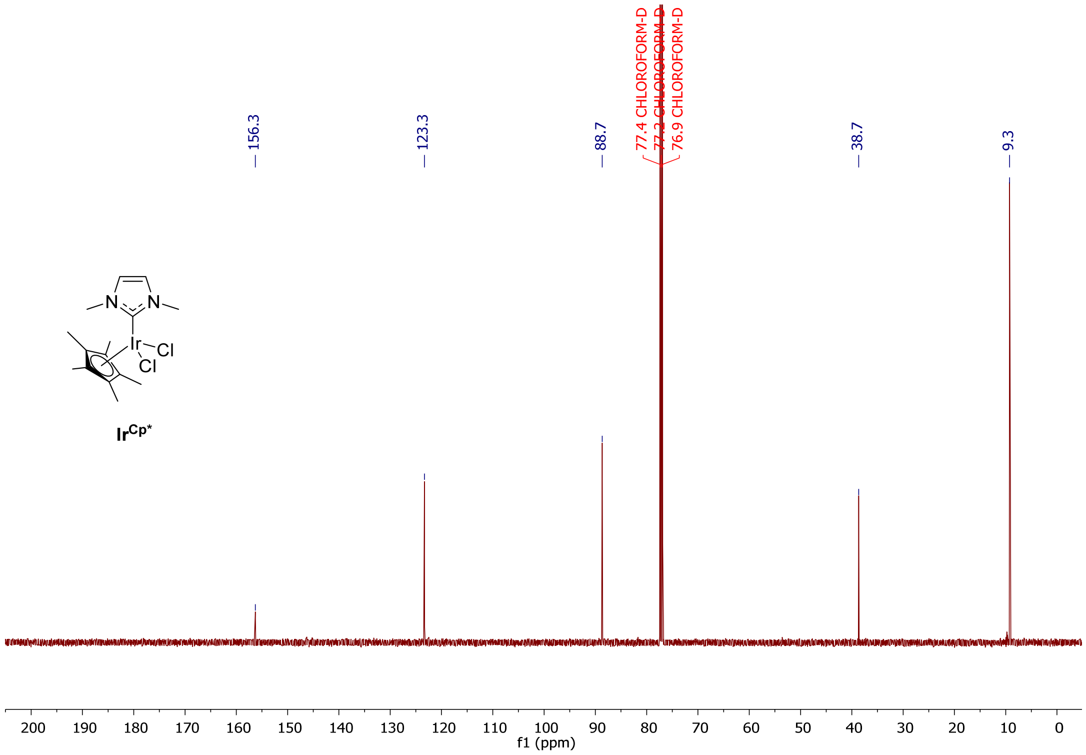

Fig. S4. ^{13}C{^{1}H} NMR spectrum of complex **Ir^{Cp*}**, recorded in chloroform-*d* at 126 MHz.

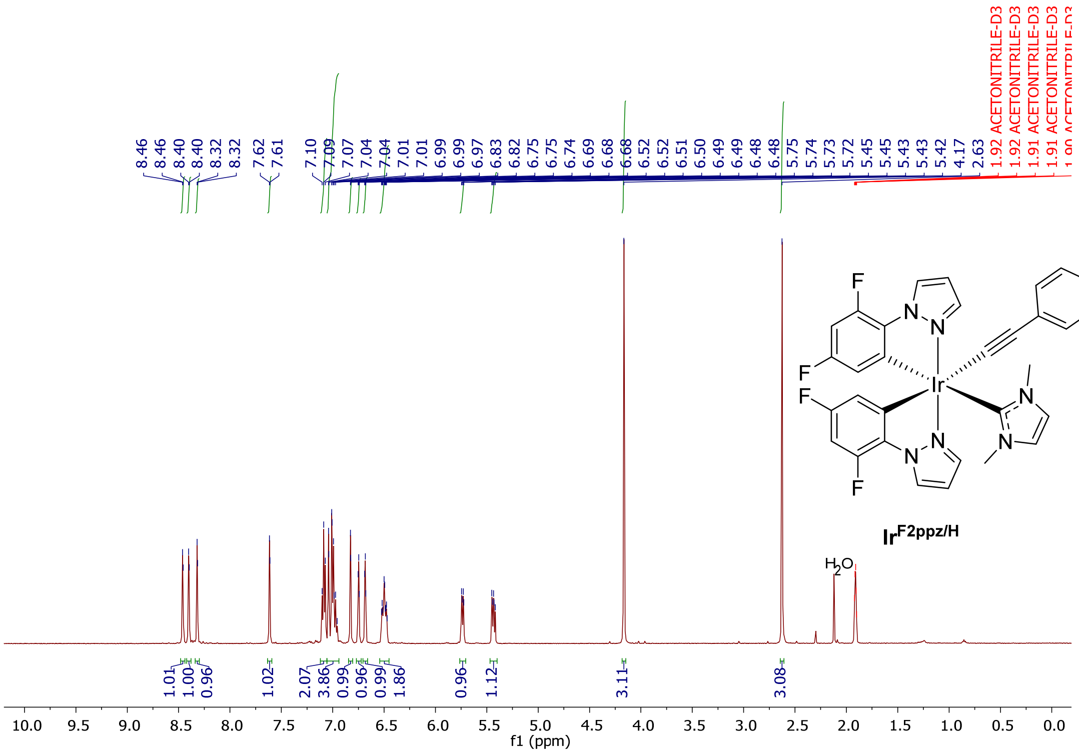

Fig. S5. ^{1}H NMR spectrum of complex **Ir$^{\mathbf{F2ppz/H}}$**, recorded in acetonitrile-*d3* at 500 MHz.

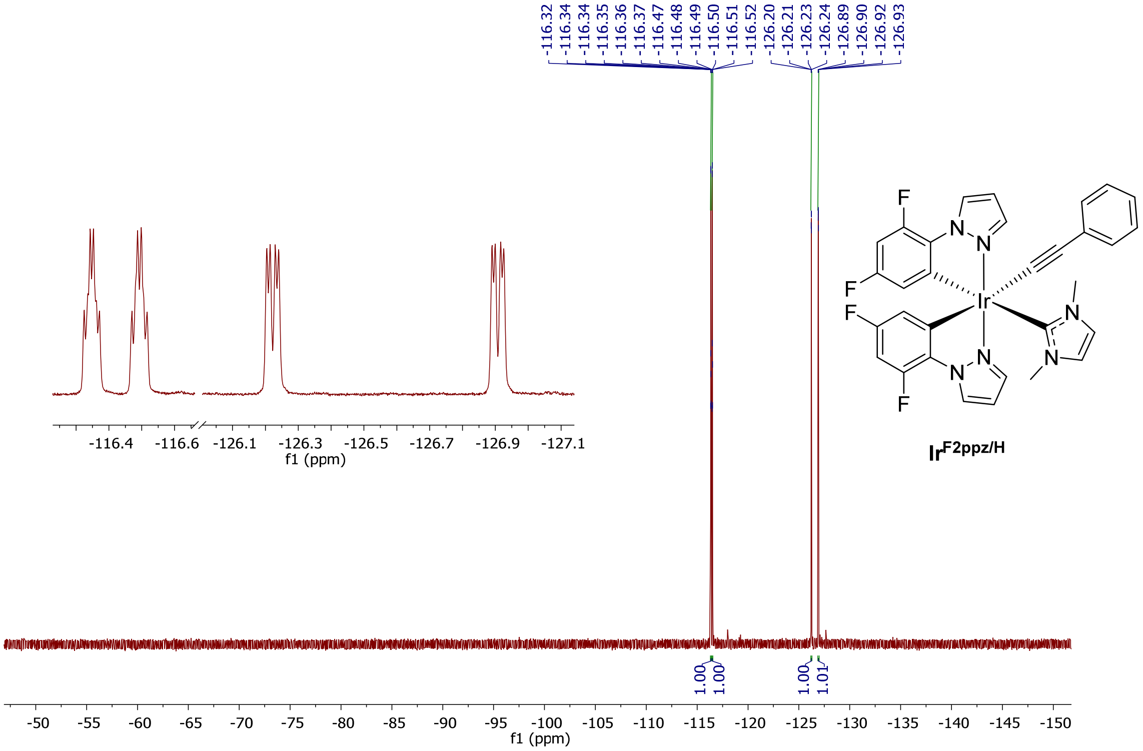

Fig. S6. ^{19}F NMR spectrum of complex **Ir$^{\mathbf{F2ppz/H}}$**, recorded in acetonitrile-*d3* at 470 MHz.

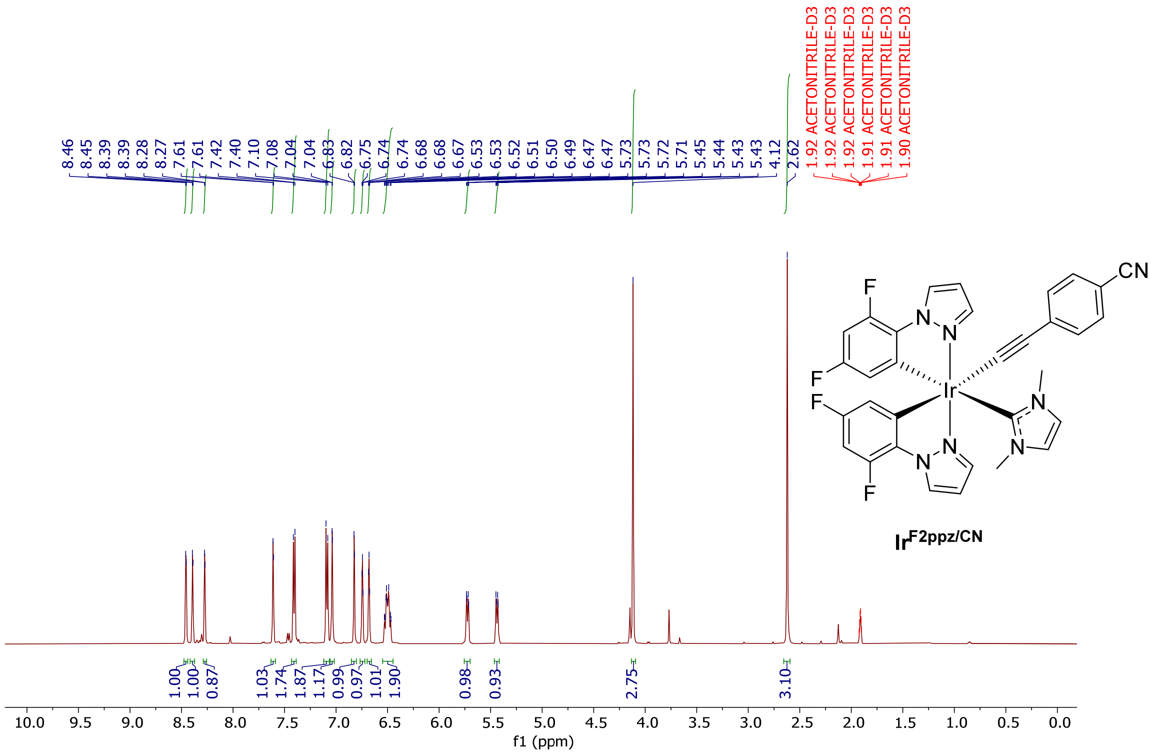

Fig. S7. ^{1}H NMR spectrum of complex **Ir$^{F2ppz/CN}$**, recorded in acetonitrile-*d3* at 500 MHz.

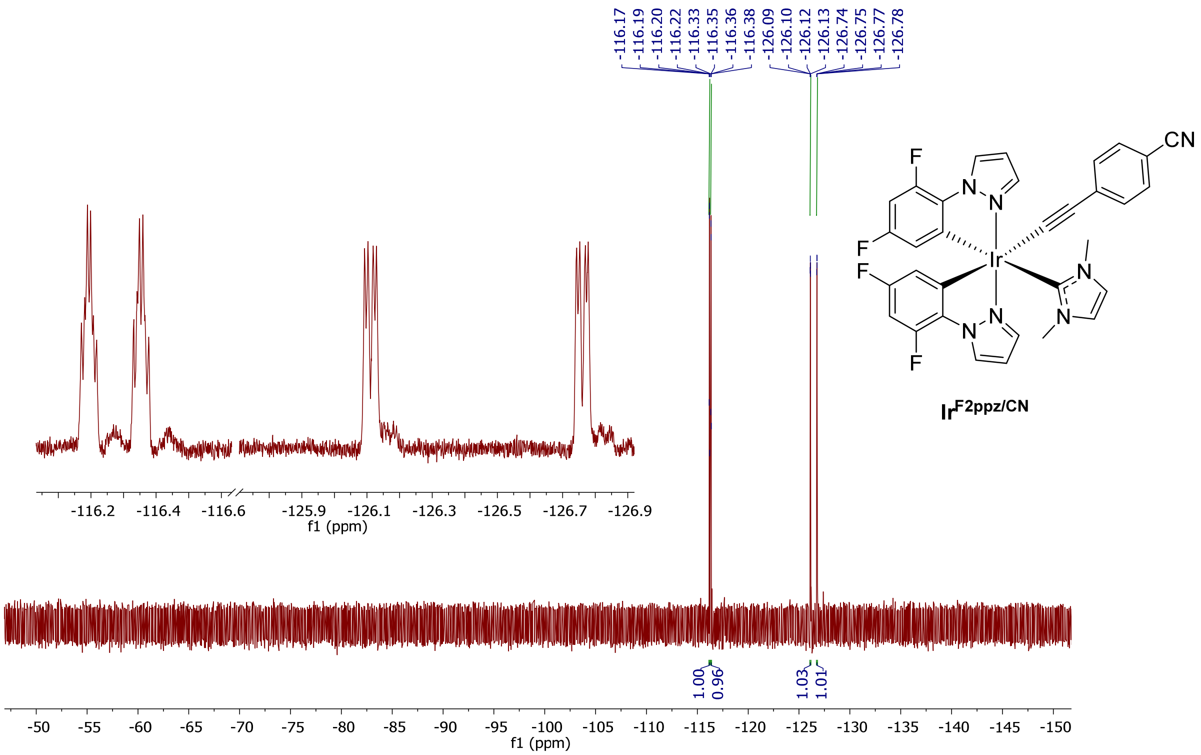

Fig. S8. ^{19}F NMR spectrum of complex **Ir$^{F2ppz/CN}$**, recorded in acetonitrile-*d3* at 470 MHz.

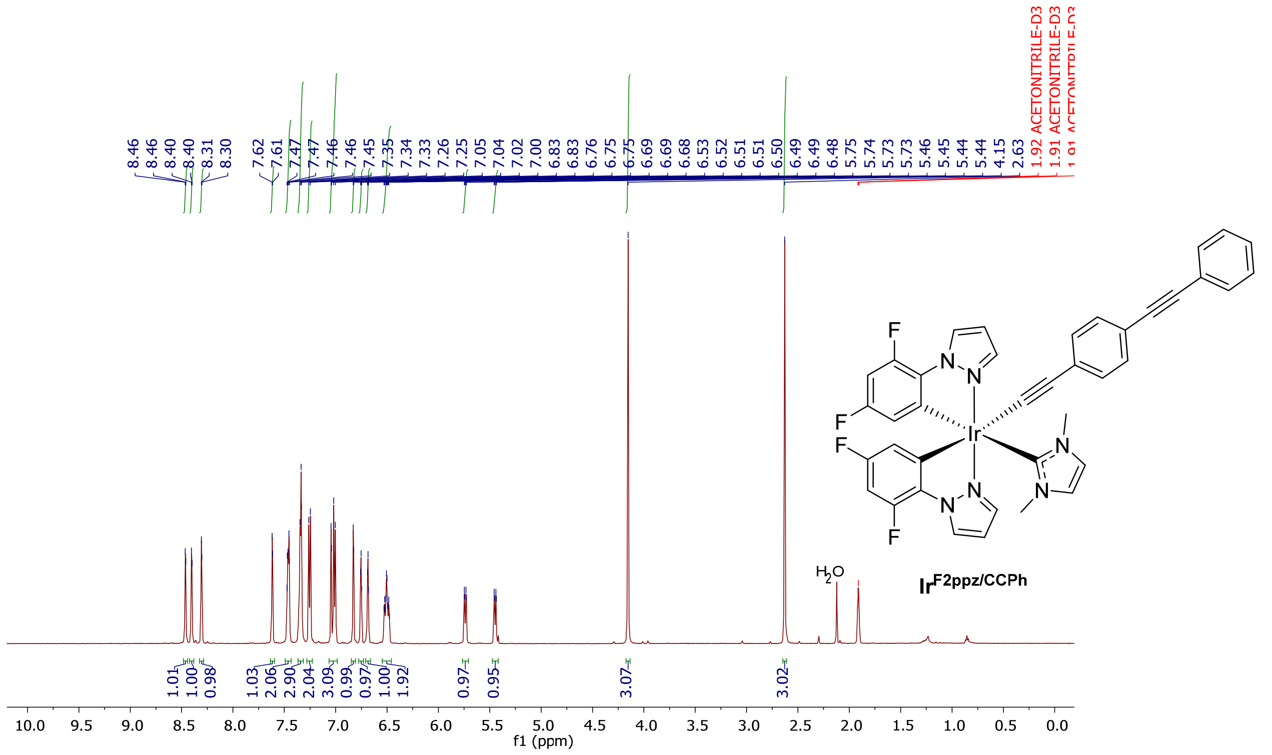

Fig. S9. ^{1}H NMR spectrum of complex **Ir$^{\mathbf{F2ppz/CCPh}}$** recorded in acetonitrile-*d3* at 500 MHz.

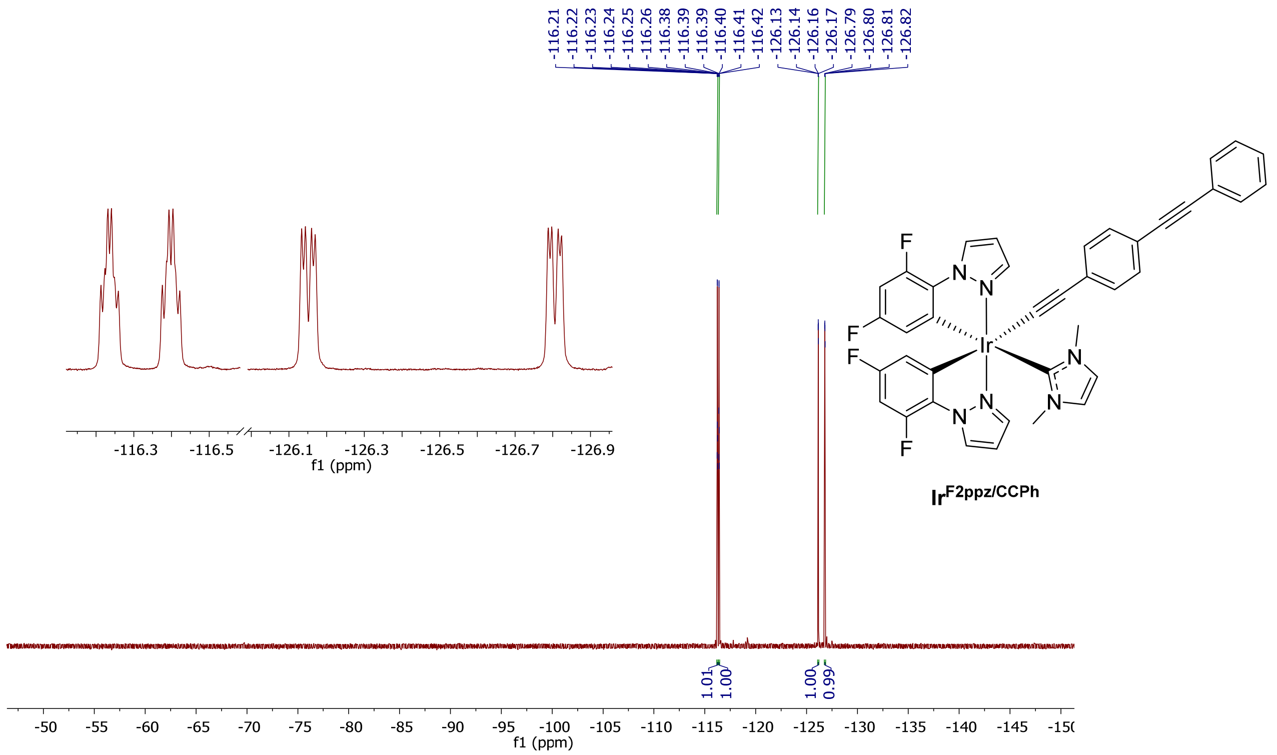

Fig. S10. ^{19}F NMR spectrum of complex **Ir$^{\mathbf{F2ppz/CCPh}}$**, recorded in acetonitrile-*d3* at 470 MHz.

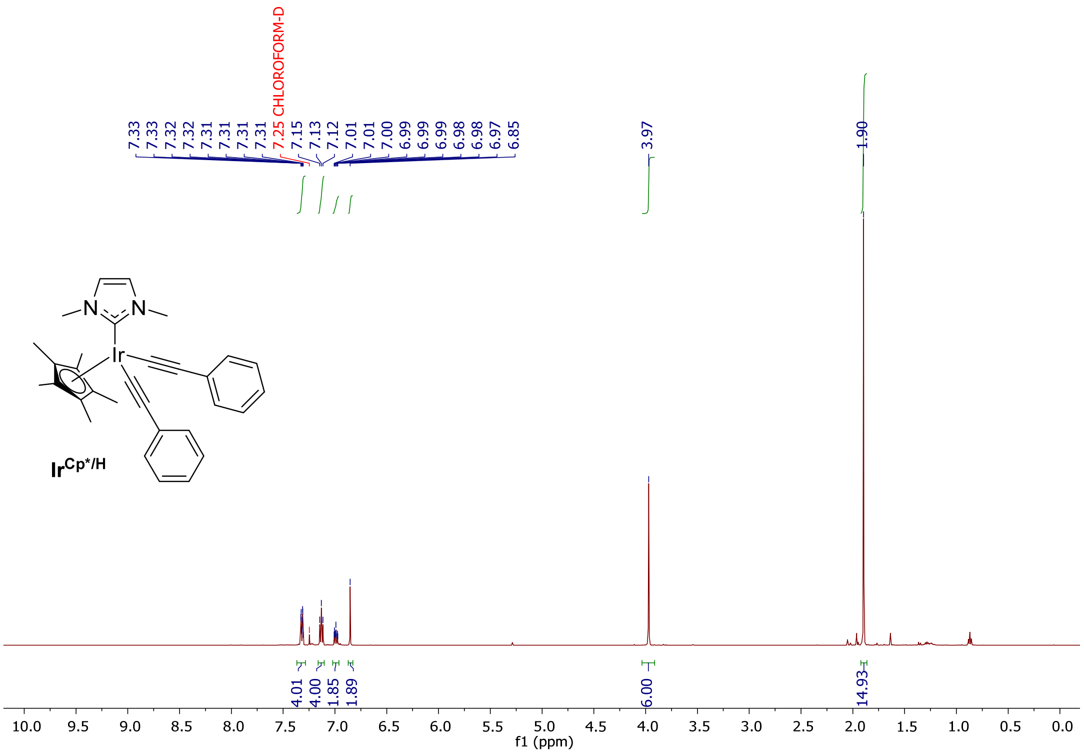

Fig. S11. ^{1}H NMR spectrum of complex **Ir$^{Cp*/H}$**, recorded in chloroform-*d* at 500 MHz.

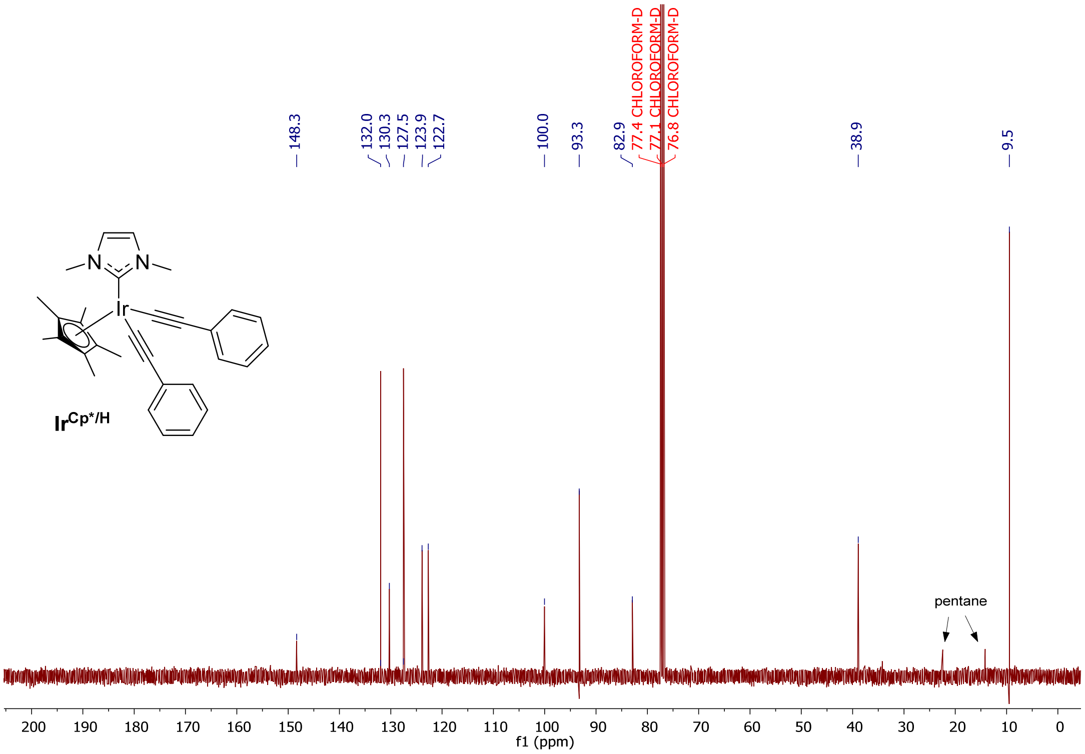

Fig. S12. ^{13}C NMR spectrum of complex **Ir$^{Cp*/H}$**, recorded in chloroform-*d* at 101 MHz.

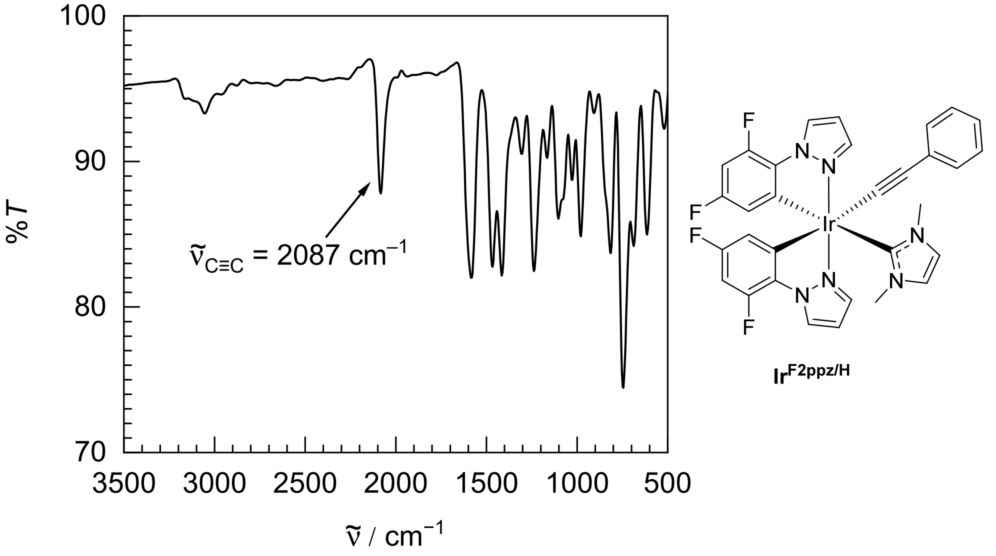

Fig. S13. FT-IR spectrum of complex **Ir$^{F2ppz/H}$**, recorded as a neat powder.

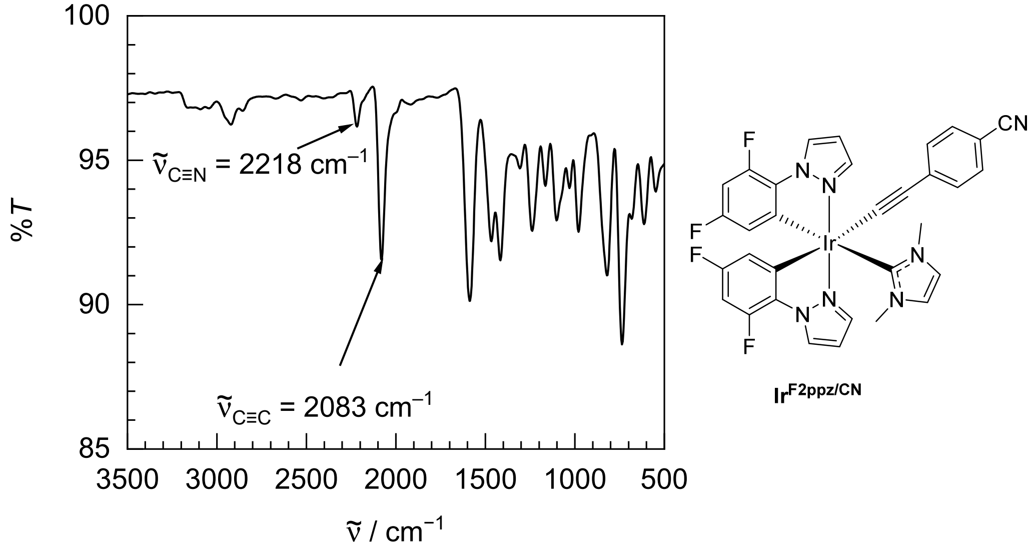

Fig. S14. FT-IR spectrum of complex **Ir$^{F2ppz/ArCN}$**, recorded as a neat powder.

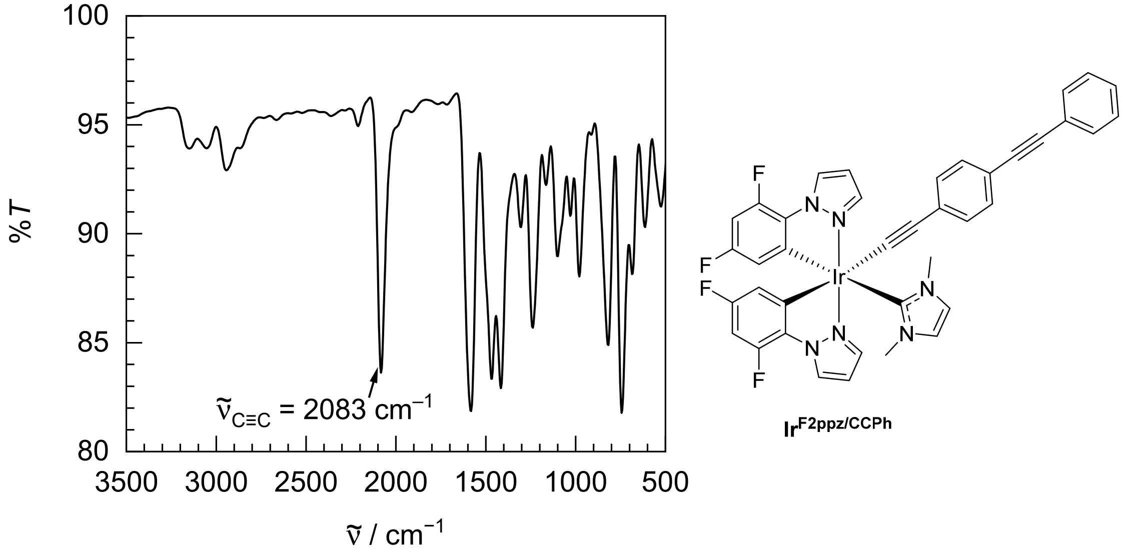

Fig. S15. FT-IR spectrum of complex **Ir**$^{\mathbf{F2ppz/CCPh}}$, recorded as a neat powder.

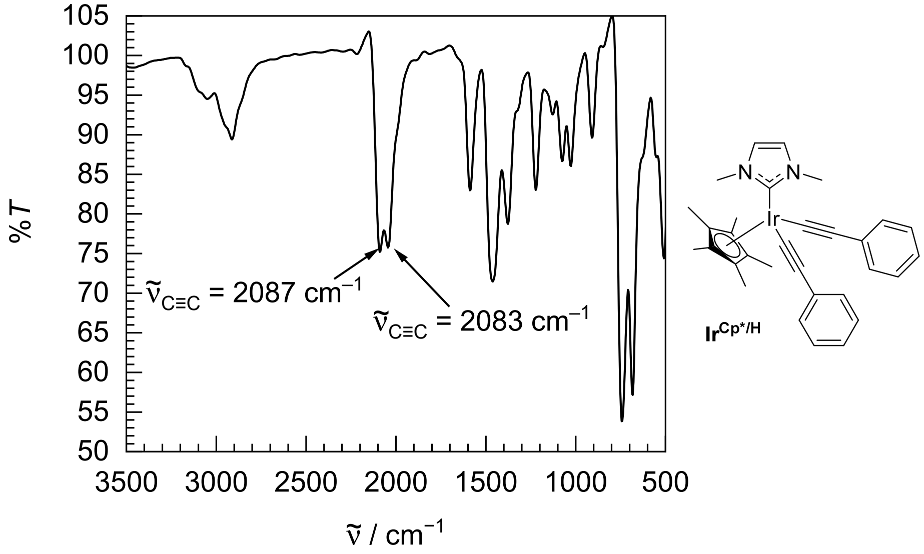

Fig. S16. FT-IR spectrum of complex **Ir**$^{\mathbf{Cp*/H}}$, recorded as a neat powder.

Results Acquired by The University of Texas at Austin Mass Spectrometry Facility

**Data File**	MSF25-0039(TS245)_hrESIpos1.d	**Sample Name**	0039(TS245)	**Comment**	0039(TS245)
**Position**	P1-E7	**Instrument Name**	6530	**User Name**	
**Acq Method**	FIA_pos.m	**Acquired Time**	8/19/2025 2:11:36 PM	**DA Method**	MSF.m

MS Zoomed Spectrum

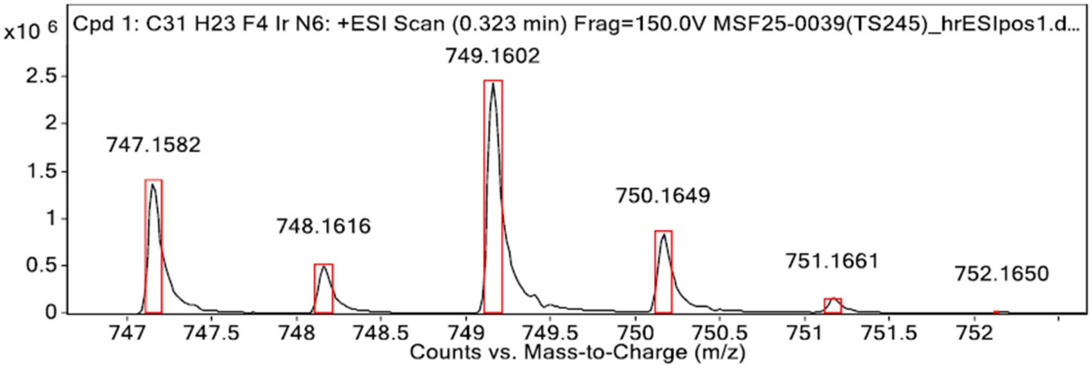

MS Spectrum Peak List

Obs. m/z	Calc. m/z	Charge	Abundance	Formula	Ion Species	Tgt Mass Error (ppm)
647.1136			3884978			
747.1582	747.1599	1	1386060	C31H23F4IrN6	(M+H)+	2.34
748.1616	748.1629	1	523526	C31H23F4IrN6	(M+H)+	1.73
749.1602	749.1624	1	2447418	C31H23F4IrN6	(M+H)+	2.85
750.1649	750.1653	1	847451	C31H23F4IrN6	(M+H)+	0.45
751.1661	751.1682	1	154650	C31H23F4IrN6	(M+H)+	2.85
753.1529	753.1741	1	23585	C31H23F4IrN6	(M+H)+	28.16

--- End Of Report ---

Fig. S17. ESI-MS accurate mass report of **Ir$^{\mathbf{F2ppz/H}}$**.

Results Acquired by The University of Texas at Austin Mass Spectrometry Facility

**Data File**	MSF25-0039(TS246)_hrESIpos1.d	**Sample Name**	0039(TS246)	**Comment**	0039(TS246)
**Position**	P1-E8	**Instrument Name**	6530	**User Name**	
**Acq Method**	FIA_pos.m	**Acquired Time**	8/19/2025 2:13:40 PM	**DA Method**	MSF.m

MS Zoomed Spectrum

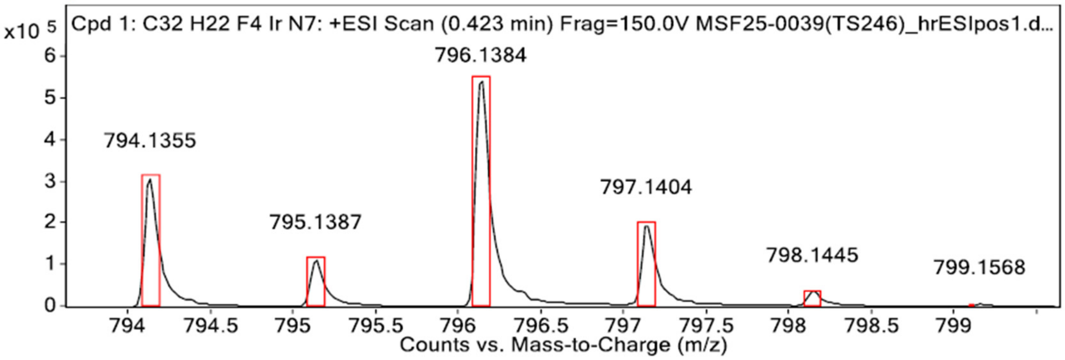

MS Spectrum Peak List

Obs. m/z	Calc. m/z	Charge	Abundance	Formula	Ion Species	Tgt Mass Error (ppm)
794.1355	794.1371	1	310627	C32H22F4IrN7	(M+Na)+	2
795.1387	795.1400	1	114550	C32H22F4IrN7	(M+Na)+	1.72
796.1384	796.1396	1	551242	C32H22F4IrN7	(M+Na)+	1.45
797.1404	797.1424	1	198924	C32H22F4IrN7	(M+Na)+	2.52
798.1445	798.1453	1	34895	C32H22F4IrN7	(M+Na)+	1.06
799.1568	799.1482	1	5674	C32H22F4IrN7	(M+Na)+	-10.75
800.1617	800.1511	1	3135	C32H22F4IrN7	(M+Na)+	-13.19

--- End Of Report ---

Fig. S18. ESI-MS accurate mass report of **Ir$^{\mathbf{F2ppz/CN}}$**.

Results Acquired by The University of Texas at Austin Mass Spectrometry Facility

**Data File**	MSF25-0039(TS240)_hrESIpos1.d	**Sample Name**	0039(TS240)	**Comment**	0039(TS240)
**Position**	P1-E6	**Instrument Name**	6530	**User Name**	
**Acq Method**	FIA_pos.m	**Acquired Time**	8/19/2025 2:09:34 PM	**DA Method**	MSF.m

MS Zoomed Spectrum

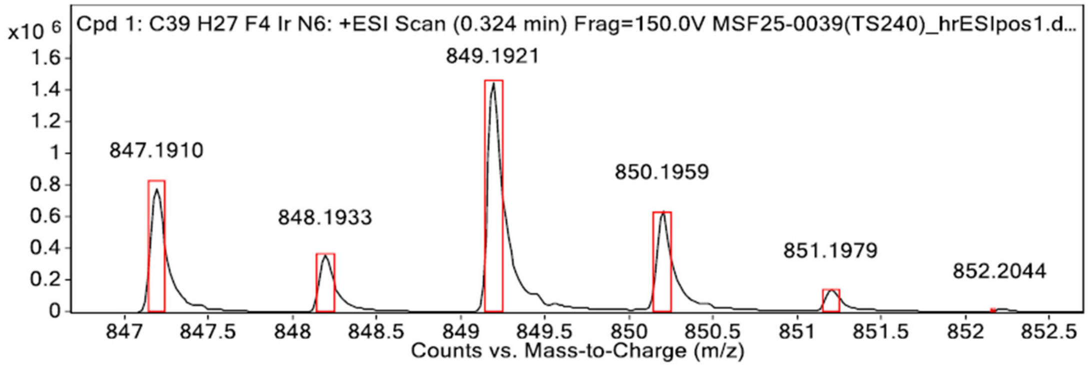

MS Spectrum Peak List

Obs. m/z	Calc. m/z	Charge	Abundance	Formula	Ion Species	Tgt Mass Error (ppm)
647.1141			1773551			
847.1910	847.1912	1	785815	C39H27F4IrN6	(M+H)+	0.26
848.1933	848.1943	1	361271	C39H27F4IrN6	(M+H)+	1.14
849.1921	849.1937	1	1459935	C39H27F4IrN6	(M+H)+	1.9
850.1959	850.1967	1	648756	C39H27F4IrN6	(M+H)+	0.91
851.1979	851.1997	1	151425	C39H27F4IrN6	(M+H)+	2.1
852.2044	852.2027	1	22228	C39H27F4IrN6	(M+H)+	-1.92

--- End Of Report ---

Fig. S19. ESI-MS accurate mass report of **Ir$^{\mathbf{F2ppz/CCPh}}$**.

Results Acquired by The University of Texas at Austin Mass Spectrometry Facility

**Data File**	MSF25-0039(TS221)_hrESIpos1.d	**Sample Name**	0039(TS221)	**Comment**	0039(TS221)
**Position**	P1-E5	**Instrument Name**	6530	**User Name**	
**Acq Method**	FIA_pos.m	**Acquired Time**	8/19/2025 2:07:31 PM	**DA Method**	MSF.m

MS Zoomed Spectrum

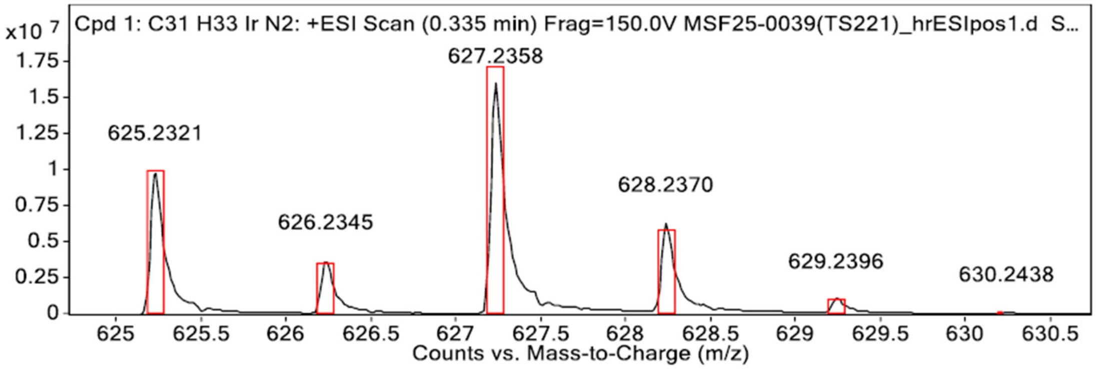

MS Spectrum Peak List

Obs. m/z	Calc. m/z	Charge	Abundance	Formula	Ion Species	Tgt Mass Error (ppm)
625.2321	625.2322	1	9944847	C31H33IrN2	(M+H)+	0.16
626.2345	626.2355	1	3751008	C31H33IrN2	(M+H)+	1.54
627.2358	627.2347	1	16124852	C31H33IrN2	(M+H)+	-1.7
628.2370	628.2379	1	6355604	C31H33IrN2	(M+H)+	1.32
629.2396	629.2411	1	1026138	C31H33IrN2	(M+H)+	2.43
630.2438	630.2443	1	119517	C31H33IrN2	(M+H)+	0.85

--- End Of Report ---

Fig. S20. ESI-MS accurate mass report of **Ir$^{\mathbf{Cp*/H}}$**.

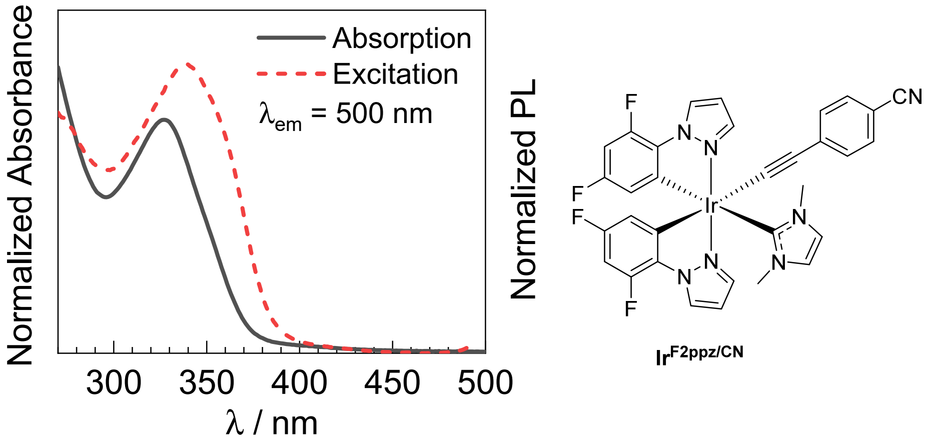

$Ir^{F2ppz/CN}$

Fig. S21. Overlaid and normalized UV–vis absorption (black solid line) and excitation (red dashed line) spectra of complex $\mathbf{Ir}^{\mathbf{F2ppz/CN}}$. The UV–vis absorption spectrum was recorded in dichloromethane and the excitation spectrum in PMMA film at 2 wt%, both at room temperature.

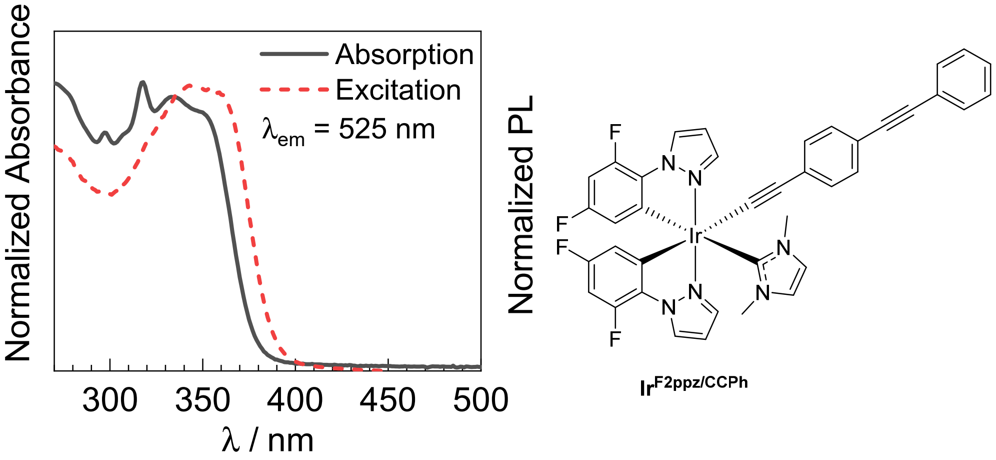

$Ir^{F2ppz/CCPh}$

Fig. S22. Overlaid and normalized UV–vis absorption (black solid line) and excitation (red dashed line) spectra of complex $\mathbf{Ir}^{\mathbf{F2ppz/CCPh}}$. The UV–vis absorption spectrum was recorded in dichloromethane and the excitation spectrum in PMMA film at 2 wt%, both at room temperature.

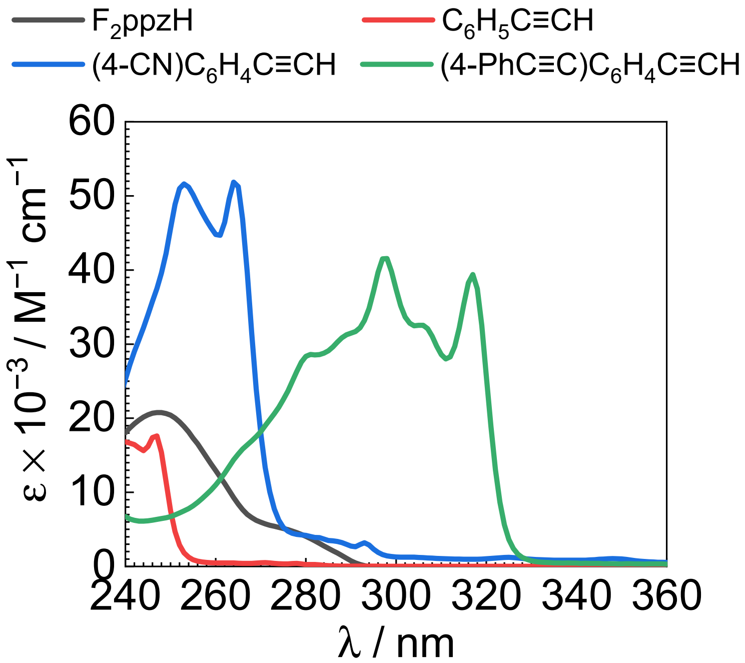

Fig. S23. Overlaid UV–vis absorption spectra of free ligands, recorded in dichloromethane at room temperature.

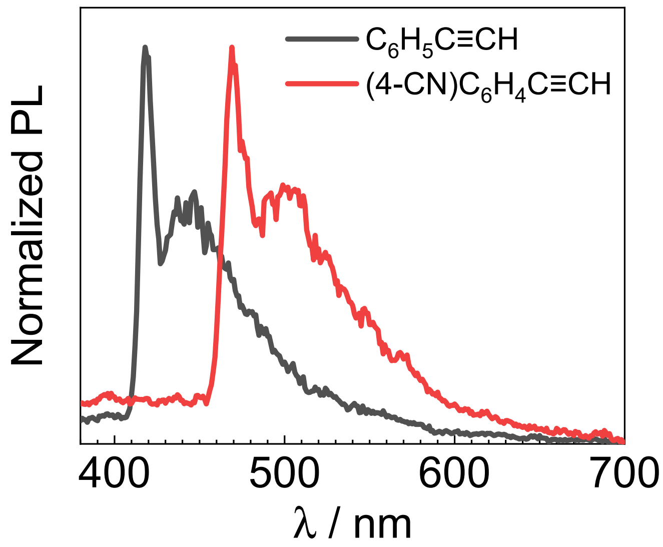

Fig. S24. Overlaid PL spectra of phenylacetylene and 4-cyanophenylacetylene, recorded in dichloromethane at 77 K. The weak signal and poor signal-to-noise ratio results in the baselines not being completely flat in both spectra.

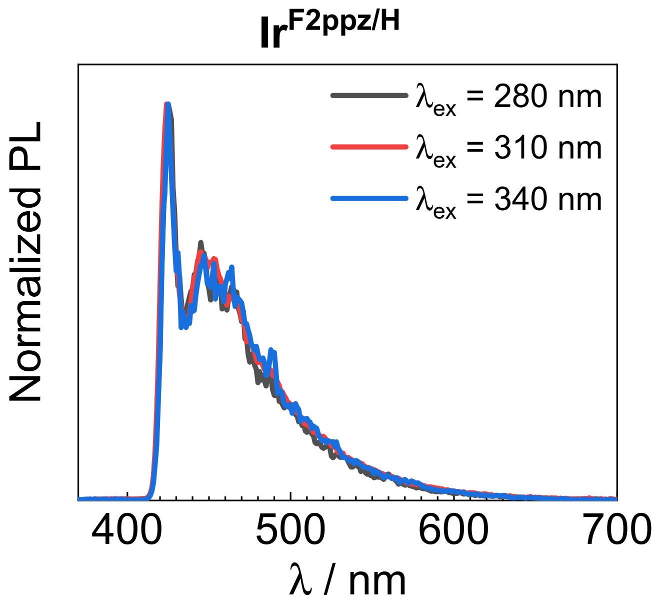

Fig. S25. Overlaid PL spectra of **Ir$^{\mathbf{F2ppz/H}}$**, recorded in dichloromethane at 77 K and different excitation wavelengths.

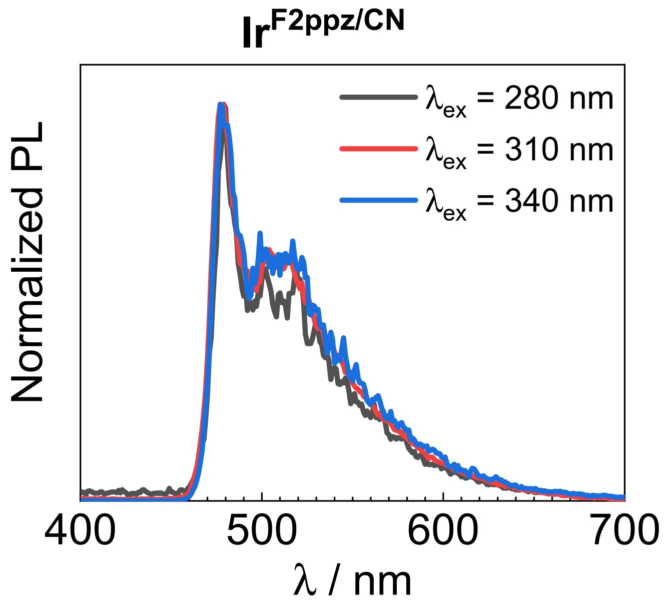

Fig. S26. Overlaid PL spectra of **Ir$^{\mathbf{F2ppz/CN}}$**, recorded in dichloromethane at 77 K and different excitation wavelengths.

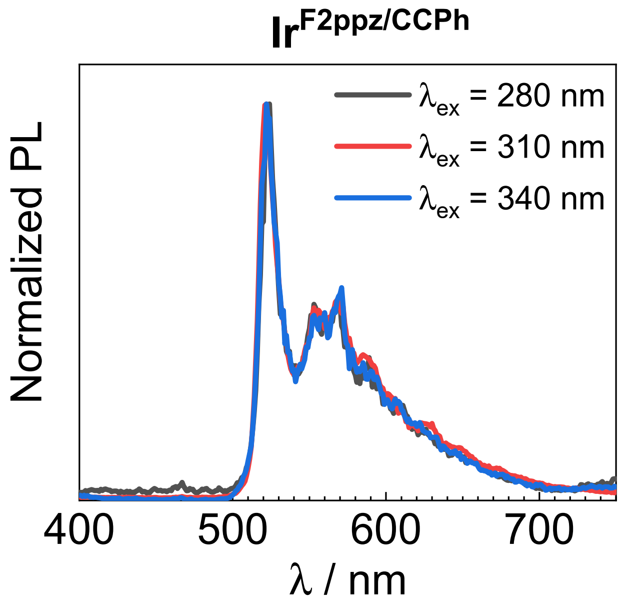

Fig. S27. Overlaid PL spectra of $\mathbf{Ir^{F2ppz/CCPh}}$, recorded in dichloromethane at 77 K and different excitation wavelengths.

Table S2. Calculated HOMO and LUMO energies for complexes **Ir$^{F2ppz/H}$** and **Ir$^{F2ppz/CN}$**, computed in the gas phase.

Compound	HOMO<br>*E* / eV	LUMO<br>*E* / eV	HOMO–LUMO gap<br>*E* / eV (λ / nm)
**Ir$^{F2ppz/H}$**	–0.238	4.114	4.352 (285)
**Ir$^{F2ppz/CN}$**	–0.253	4.003	4.256 (291)

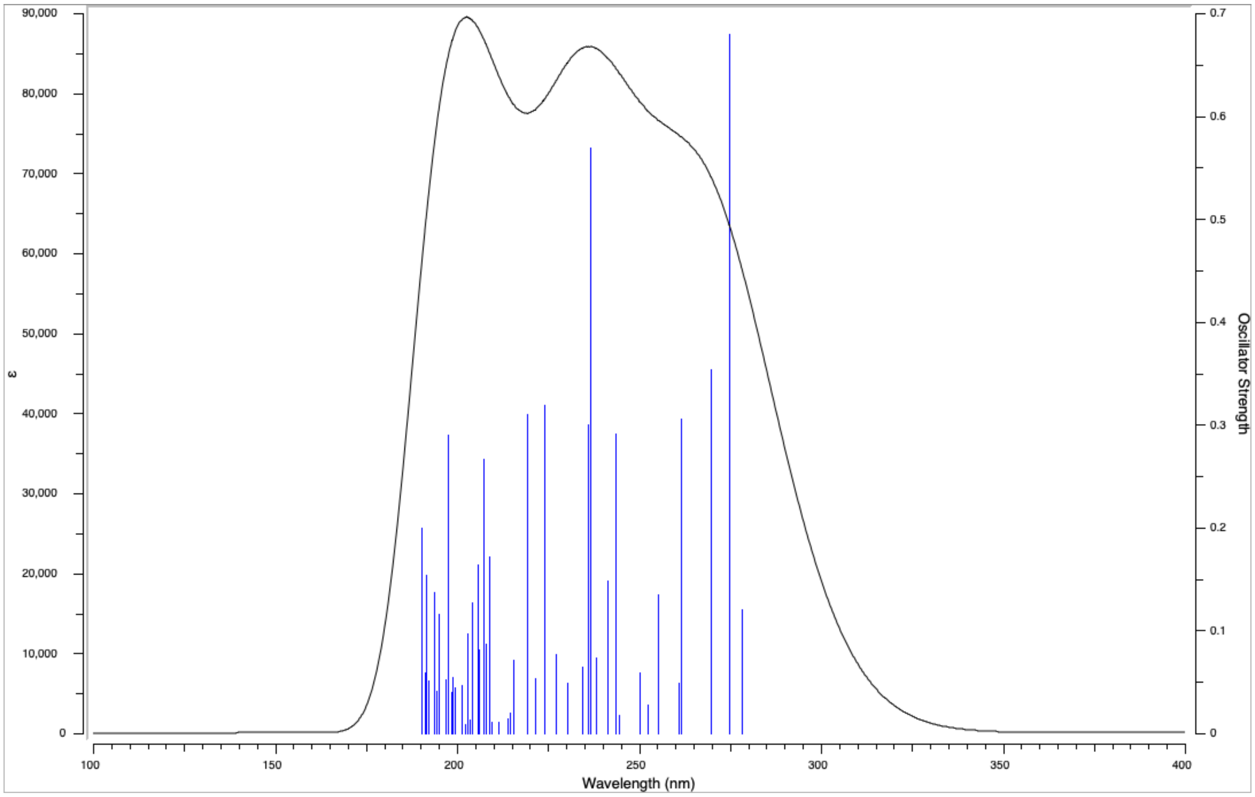

Fig. S28. Simulated UV–vis absorption spectrum of **Ir$^{F2ppz/H}$**, computed via TD-DFT with CH_2Cl_2 implicit solvation (SMD model).

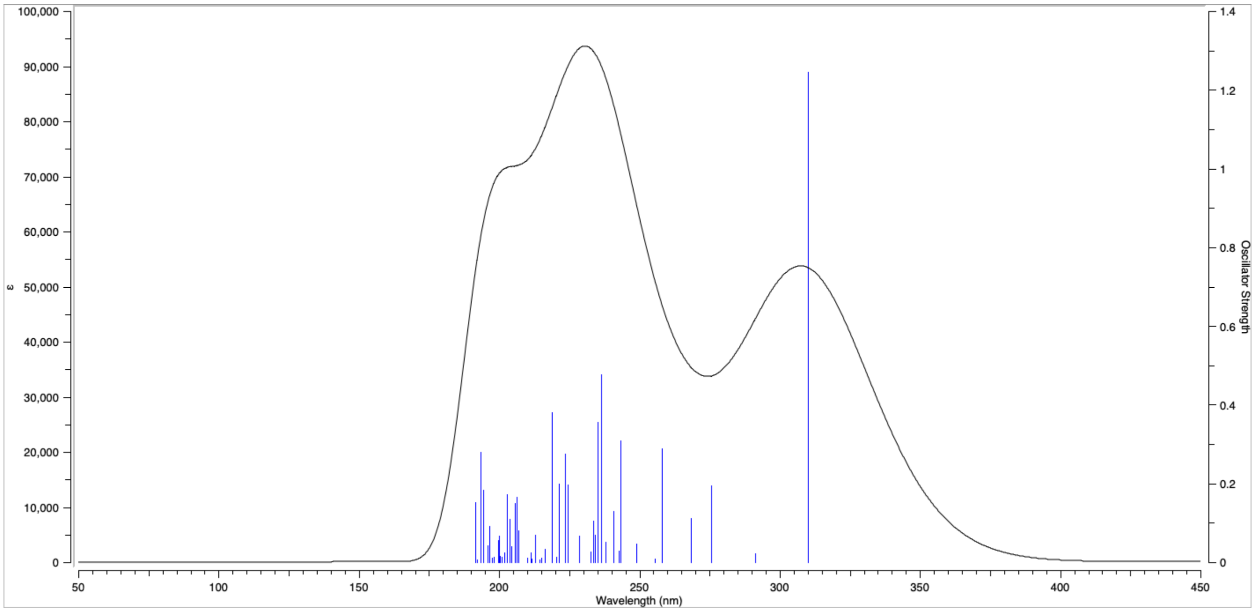

Fig. S29. Simulated UV–vis absorption spectrum of **Ir$^{\mathbf{F2ppz/CN}}$**, computed via TD-DFT with CH_2Cl_2 implicit solvation (SMD model).

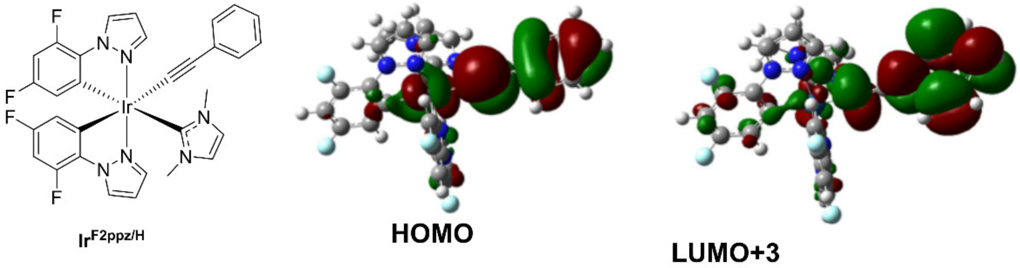

Fig. S30. Orbitals involved in the dominant transition of the $S_0 \rightarrow T_1$ excitation of **Ir$^{\mathbf{F2ppz/H}}$**.

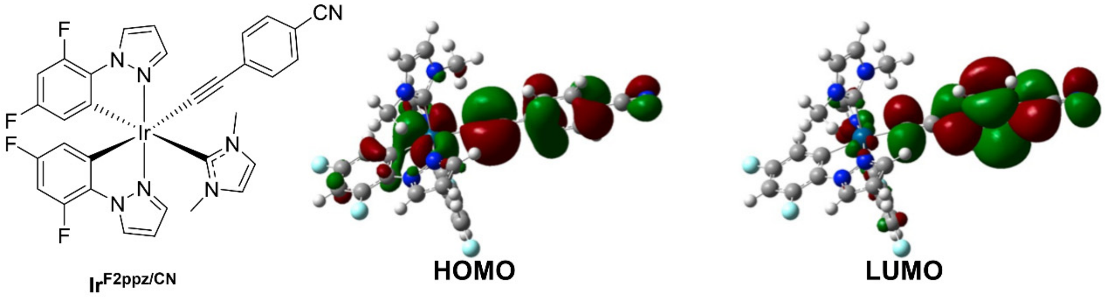

Fig. S31. Orbitals involved in the dominant transition of the $S_0 \rightarrow T_1$ excitation of **Ir$^{\mathbf{F2ppz/CN}}$**.

Cartesian coordinates of the optimized geometries

Ir$^{F_2ppz/H}$

Ir 0.13738635 -0.30221642 -0.19159465

F 4.39453593 -0.69627314 2.87177775

F 0.33943138 4.70268904 -1.78702558

N 0.37054117 -0.85360447 1.74690540

F 0.27889642 4.23927044 2.85958781

F 5.44500031 0.16175120 -1.59643513

N 0.20769166 0.53434572 -2.05576428

N 0.30194986 1.87821821 -2.08627731

N -1.08659706 -2.73934168 -1.75484046

C -0.15079781 -2.30318251 -0.86978688

N 1.63290712 -0.81418656 2.22325419

C 0.24655759 1.69551656 0.28614060

C 2.20933237 -0.33491307 -0.04184114

N 0.48816529 -3.44446642 -0.50169052

C -1.89928209 -0.15316147 0.02862227

C 0.28746187 2.53575938 -0.83201819

C -2.09997731 -1.95753145 -2.44349754

H -3.08610443 -2.22383311 -2.06805727

H -2.04386517 -2.16194186 -3.51258009

H -1.95007941 -0.90428167 -2.25409891

C 2.65107635 -0.53567380 1.27652345

C -0.42650276 -1.16155693 2.75712869

H -1.48840116 -1.21417271 2.58682347

C 0.24348268 2.30119414 1.53818560

H 0.19542571 1.71436766 2.44566129

C 3.18847378 -0.09748080 -1.00305596

H 2.92314458 0.07065787 -2.03897841

C 0.34636871 0.11014160 -3.30419978

H 0.32239923 -0.94377362 -3.51639463

C 1.57693772 -3.54539082 0.45011002

H 2.37392531 -2.85965587 0.18183884

H 1.96001873 -4.56225348 0.42654031

H 1.22867156 -3.32424199 1.45703574

C -1.02926228 -4.10468995 -1.91980029

H -1.69535450 -4.62881951 -2.58048776

C 0.52106447 1.19572774 -4.16182205

H 0.65276172 1.17673503 -5.22805257

C 1.63665642 -1.10906459 3.53995595

H 2.55014934 -1.12816186 4.10203482

C 3.99375898 -0.49794671 1.60231048

C -3.09024448 -0.07577985 0.25880948

C 0.31372032 3.91215815 -0.69786538

C 0.33360113 -1.33830741 3.91590014

H -0.02009259 -1.58749702 4.89958896

C 0.49030041 2.30540653 -3.35011750

H 0.59122421 3.34981100 -3.57285463

C -0.03967565 -4.54942207 -1.12959638

H 0.33697869 -5.54155976 -0.96104951

C 0.31452761 4.51183996 0.54245841

H 0.33713748 5.58599806 0.64145918

C 4.52271919 -0.06669979 -0.64954224

C 0.28052899 3.67592063 1.64226578

C 4.95969401 -0.26265979 0.64605175

H 6.00584776 -0.23053262 0.90815813

C -5.26437406 -1.13928889 0.69481186

H -4.77463725 -2.10421964 0.67218197

C -4.49543793 0.01284938 0.49734400

C -7.25570643 0.18326874 0.95327961

H -8.32109337 0.24984751 1.12963226

C -6.62793483 -1.05335167 0.92004833

H -7.20402084 -1.95728115 1.07154241

C -5.14148217 1.25317007 0.53559987

H -4.55545701 2.15014910 0.38779484

C -6.50483651 1.33441138 0.76031209

H -6.98519938 2.30405682 0.78650620

Ir$^{F_2ppz/CN}$

Ir 0.45321562 -0.31909312 -0.20741912

F 4.53408742 -0.50938714 3.10402989

F 0.57955452 4.67753346 -1.83630604

N 0.59421660 0.84896048 1.74731990

F 0.24709085 4.24769338 2.80155804

F 5.80739433 0.35869625 -1.30338569

N 0.60840117 0.50561027 -2.07245987

N 0.65891029 1.85177884 -2.10996432

N -0.57071046 -2.81567565 -1.82070335

C 0.28921830 -2.33634329 -0.88252833

N 1.82354027 -0.75346882 2.29620099

C 0.46219800 1.68588293 0.25635950

C 2.51181065 -0.26652381 0.06460424

N 0.95061904 -3.44745473 -0.46650253

C -1.59295641 -0.23652749 -0.11094708

C 0.54321595 2.51800800 -0.86541554

C -1.57097246 -2.07902510 -2.57464025

H -2.56672073 -2.38101637 -2.25582410

H -1.44541263 -2.28847931 -3.63664191

H -1.47047781 -1.01909671 -2.38886211

C 2.88303293 -0.43795074 1.40815156

C -0.24621410 -1.18634144 2.71224135

H -1.29322153 -1.28687118 2.48306990

C 0.36031737 2.30000135 1.49978567

H 0.27758606 1.71887176 2.40844074

C 3.53385395 0.00589193 -0.84049323

H 3.32333552 0.15471187 -1.89177355

C 0.84079468 0.07695350 -3.30554252

H 0.86759822 -0.97865952 -3.50882728

C 1.98044652 -3.49841415 0.55283986

H 2.75566070 -2.77052580 0.33769132

H 2.41697845 -4.49368925 0.55023237

H 1.55757312 -3.30245906 1.53624036

C -0.44629696 -4.17830942 -1.97171587

H -1.04639847 -4.73351641 -2.66924981

C 1.03261090 1.16125097 -4.16069377

H 1.23267656 1.13877916 -5.21616134

C 1.76312094 -1.04197267 3.61281563

H 2.64225489 -1.01990433 4.22715673

C 4.20140437 -0.33891794 1.81173310

C -2.79709683 -0.17914856 0.05230408

C 0.51362380 3.89562486 -0.74337711

C 0.45123551 -1.32502561 3.91424764

H 0.05171567 -1.58473954 4.87744391

C 0.91279029 2.27538766 -3.36336062

H 0.99253155 3.32084510 -3.58987308

C 0.50964833 -4.57663906 -1.11787435

H 0.91649913 -5.55113263 -0.91924617

C 0.41670471 4.50418778 0.48880926

H 0.39651202 5.57923702 0.57853070

C 4.84261303 0.09752496 -0.40984165

C 0.34349699 3.67628443 1.59266631

C 5.21049731 -0.06900394 0.91102720

H 6.23711796 0.01088401 1.23332059

C -4.96793839 -1.25281668 0.46514606

H -4.46429289 -2.20707821 0.53800063

C -4.20794522 -0.10234493 0.21622804

C -6.98623651 0.04845337 0.52743399

C -6.33711150 -1.18198046 0.61923278

H -6.91294197 -2.07682080 0.81163305

C -4.87511331 1.12670965 0.12986115

H -4.29795907 2.02130483 -0.05725624

C -6.24381253 1.20351392 0.28208364

H -6.74771045 2.15775059 0.21338129

C -8.40655963 0.12400440 0.68436208

N -9.54782487 0.18196842 0.80950400

References

1 T.-Y. Li, X. Liang, L. Zhou, C. Wu, S. Zhang, X. Liu, G.-Z. Lu, L.-S. Xue, Y.-X. Zheng and J.-L. Zuo, *Inorg. Chem.*, 2015, **54**, 161–173.

2 K.-Y. Zhao, G.-G. Shan, Q. Fu and Z.-M. Su, *Organometallics*, 2016, **35**, 3996–4001.

3 Y. Tanabe, F. Hanasaka, K. Fujita and R. Yamaguchi, *Organometallics*, 2007, **26**, 4618–4626.

4 N. Schwarz, X. Sun, R. Yadav, R. Köppe, T. Simler and P. W. Roesky, *Chem. – Eur. J.*, 2021, **27**, 12857–12865.
